# Sex representation in trials relative to indication-specific disease burden in FDA-approved drugs (2015–2023)

**DOI:** 10.1038/s41467-026-74469-z

**Published:** 2026-06-23

**Authors:** Sophie Zaaijer, Simon C. Groen

**Affiliations:** 1 Division of Hematology and Oncology, Department of Medicine, University of California Irvine School of Medicine, Irvine, CA USA; 2 Department of Nematology, Institute for Integrative Genome Biology, University of California Riverside, Riverside, CA USA

**Keywords:** Drug safety, Disease genetics, Clinical trials, Signs and symptoms

## Abstract

Sex-based disparities in disease burden and therapeutic response motivate efforts to prioritize women’s health in drug development. We analyzed 195 drugs across 98 indications approved by the United States Food and Drug Administration (FDA) between 2015 and 2023 to assess whether industry focus and clinical trial enrollment reflect disease prevalence among males and females. Here, we show that therapies for female-predominant indications receive 1.5 times more approvals than male-predominant indications. Additionally, among trials leading to approval, female participation aligns with or exceeds disease prevalence in 67% of cases. Alignment is strongest in oncology, whereas cardiovascular and autoimmune diseases most often under-enroll women, with no improvement over time. We discuss that gains in female enrollment have plateaued and that further progress will require advances in diagnostics, greater use of objective endpoints, and improved tools to assess risks for women of reproductive potential.

## Abstract (web summary)

Analysis of clinical trial cohorts for 218 FDA-approved drugs finds that, although women are now often adequately represented in trials, progress has plateaued and gaps persist in specific disease areas; the study outlines actionable next steps.

## Introduction

Women have been systematically underrepresented in clinical trial cohorts for much of the 20^th^ century. This broad exclusion—justified by concerns about fetal risks for pregnant women, menstrual/hormonal variability, and potential interactions with contraceptives—entrenched a male-default paradigm in clinical trial design. Since 1993, policy shifts including the US National Institutes of Health (NIH) Revitalization Act, subsequent FDA guidance^1^, and the FDA Drug Trial Snapshots Program (DTSP) have aimed to redress this imbalance. Current frameworks enable risk-mitigated inclusion of women of reproductive potential. In practice, three pillars govern safe participation of women in clinical trials: (i) nonclinical (or “preclinical”) developmental and reproductive toxicity (DART) studies that inform dose and timing of drug administration (e.g., ICH S5[R3]); (ii) staged human exposure practices that link clinical trial enrollment to the availability of DART evidence (e.g., ICH M3[R2]); and (iii) participant-level safeguards—baseline and periodic pregnancy testing as well as documenting use of effective contraception or abstinence^1^. Together, these policy shifts acknowledge historic inequities and replace blanket exclusion of women from clinical trials with protocols for change.

Despite these reforms, reports continue to describe ongoing disparities in female representation among clinical trial cohorts within specific therapeutic areas^2–7^. Trials testing new therapies for cardiovascular, autoimmune, and psychiatric disease indications frequently under-enroll women^2–4^, even though their disease burden is equal to or greater than that of men. Such mismatches raise deeper questions: why do these disparities persist, what factors—biological, methodological or regulatory—drive them, and what are the consequences for the validity of trial outcomes?

A stringent standard for representation assesses whether clinical trial enrollment aligns with disease burden by sex for the relevant indication using prevalence or incidence as reference metrics^2,4,6^. When enrollment is misaligned, efficacy and safety outcomes from a clinical trial may be biased and recognition of sex-specific effects may therefore be delayed. In contrast, aligned trial enrollment heightens the chance that sex-biased efficacy and safety signals will be detected. The central nervous system (CNS) depressant zolpidem forms a case in point: years after official approval, the FDA lowered the recommended dose for women after pharmacokinetic studies showed they experience prolonged risks for impaired driving relative to men^8^; notably, women report insomnia ~ 1.5 × more often than men^9^. One reason for the roughly two-decade delay in taking this measure was that crash reports were too confounded—by uncertainty around dose, timing, and co-ingestants—to isolate risks attributable to the drug. Only after the data were analyzed separately by sex did the sex-biased differences in zolpidem metabolism and next-morning impairment become evident, which contributed to the FDA’s decision to revise labeling and include lower recommended doses for women^8^.

Most of the previous studies that focused on evaluating female representation in clinical trials used datasets from government-funded registries such as ClinicalTrials.gov^2,3,6,7^. These resources capture all trials including; successful and failed to interventional and non-interventional trials. By contrast, interventional pivotal trials supporting FDA approvals of new drugs are predominantly industry-sponsored. These trials represent the decisive stage of drug development, where regulatory oversight, commercial imperatives, and the fundamental question of whether patients—irrespective of sex—will benefit from treatment converge.

Therefore, in this study, we focus on pivotal trials that supported FDA approvals to evaluate progress in women’s health. We address two specific questions:

(1) To what extent does the biopharmaceutical industry prioritize female-predominant disease indications?

(2) To what extent are women—defined by the FDA as part of a binary male/female biological sex variable—proportionally represented in late-stage drug development, compared to population-level relative disease burdens among women?

Taken across therapeutic areas, this assessment shows whether contemporary industry portfolio decisions and pivotal trial designs are narrowing long-standing gaps in female representation or whether structural barriers persist.

## Results

### Data characterization

**Fig. 1:**
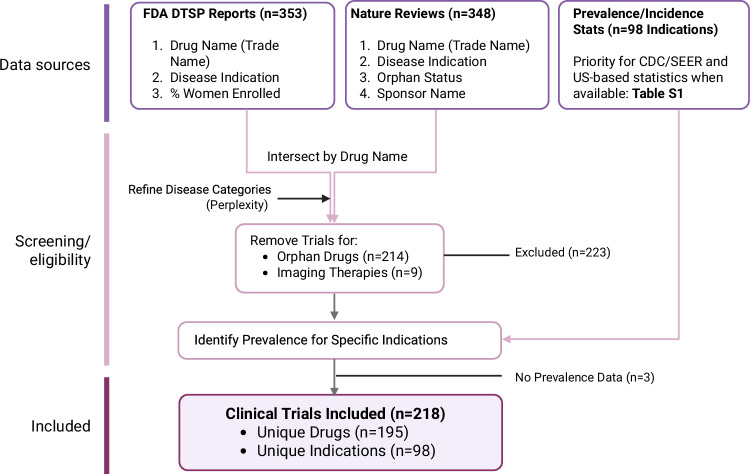
Flow diagram that outlines which clinical trials were included in our analyses. First, a total of 353 Drug Trial Snapshot Program (DTSP) reports for Phase III clinical trials that supported approval of 348 drug treatments by the US FDA Center for Drug Evaluation and Research (CDER) from 2015 to 2023 were identified. Data from 223 DTSP reports was excluded because trials were conducted for Orphan drugs or for *Imaging Therapies*—patterns in relationships between clinical trial enrollment and statistics on disease burden cannot be evaluated robustly for Orphan drugs, while trials for therapies categorized as *Imaging Therapies* are less suitable for addressing questions around sex-based biases in clinical trial enrollment patterns, respectively. It was possible to retrieve prevalence and incidence statistics for 98 disease indications, treatments for which were tested across 221 pivotal trials. Finally, we were unable to retrieve appropriate prevalence or incidence data for three disease indications, leaving data from 218 trials for analysis. Analyses included drug metadata from Nature Reviews Drug Discovery.

**Fig. 2:**
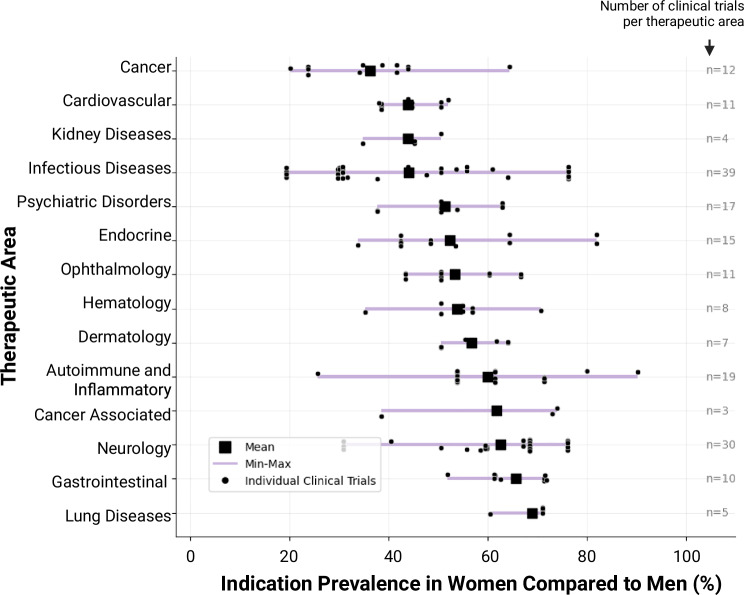
Prevalence of disease indications within therapeutic areas (excluding sex-specific indications). Black squares indicate the mean prevalence within each therapeutic area. Individual dots represent the prevalence of individual disease indications. Vertical lines indicate the range (minimum–maximum) of prevalence values within each therapeutic area. The category “Cancer-associated” includes indications such as chemotherapy-induced emesis, nausea and vomiting associated with chemotherapy, and chemotherapy-induced myelosuppression. A full list of disease indications is provided in Supplementary Data 2. Source data are provided with this paper.

We analyzed a total of 98 specific disease indications (falling under 14 therapeutic areas) for which drugs were developed that attained FDA approval between 2015 and 2023 (Fig. 1). Each therapeutic area showed a wide variation in disease prevalence ratios between men and women (as derived from disease prevalence and incidence data, see Methods) across individual indications (Fig. 2 and Supplementary Data 2). Sex-specific cancer indications (e.g., prostate, breast, ovarian, and cervical cancer) are categorized under the therapeutic area *Cancer* and are included to characterize the broader landscape of attention, even though they do not permit direct comparison of representation in clinical trials between the sexes. Among indications affecting both sexes, the next area with the highest mean relative female prevalence is *Lung Disease* at 69% (*n* = 5), which includes asthma and chronic obstructive pulmonary disease (COPD), followed by *Gastrointestinal Disorder* indications at 66% (*n* = 10), including irritable bowel syndrome and chronic idiopathic constipation.

### Commercial drug development efforts do target female-predominant disease indications

**Fig. 3:**
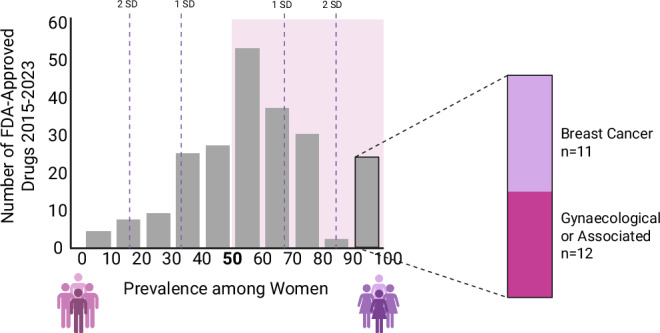
Number of disease indications within each prevalence category, including sex-specific indications (see Supplementary Data 3 for the sex-specific exclusion table). Indications were binned by prevalence ratios among women. The following five categories are indicated: (1) highly male-biased indications (their prevalence among females was at least 2 standard deviations [SDs] lower than the US Census mean of 50.5%); 2) male-biased indications (≤ 1 SD lower than the mean); (3) sex-neutral indications (within ± 1 SD of the mean); (4) female-biased indications (≥ 1 SD higher than the mean); (5) highly female-biased indications (≥ 2 SDs higher than the mean). Created in BioRender. Zaaijer, S. (2026) https://BioRender.com/z0nr14z. Source data are provided in this paper.

To address our first key question, we assessed industry investment in therapies for (a) sex-specific and (b) female-predominant diseases (diseases with higher relative prevalence among women). Among 195 FDA-approved drugs, 12 targeted gynecological conditions (e.g., breast, ovarian, and cervical cancer, postpartum depression, endometriosis associated; see comprehensive list in Supplementary Data 2), and four addressed andrological indications such as prostate cancer. In addition, 11 new drugs were approved to treat subtypes of breast cancer, a disease affecting women in ~ 99% of cases (Fig. 3). Together, between 2015 and 2023, women-specific indications received 5.5-fold higher investment compared with male-specific indications.

Indications affecting both men and women relatively equally—defined as sex-based prevalence within ± 1 SD of the US Census mean for the proportion of women in the US population (50.5%) over the nine-year period—accounted for 63% of non–sex-specific approvals (*n* = 121 of 191; Fig. 3).

Diseases with markedly higher female prevalence accounted for 22% of approvals (*n* = 42 of 191). These included conditions with prevalence ≥ 2 SD ( ≥ 67% and < 83.8%) or ≥ 3 SD ( ≥ 83.8%) above the US Census mean (Supplementary Data 3).

Indications with higher male prevalence accounted for 15% of approvals (*n* = 28 of 191). Overall, female-predominant indications received ~ 1.5 times as many FDA-approved treatments as male-predominant indications (Supplementary Data 3).

### Clinical trial representation aligns with indication prevalence

**Fig. 4:**
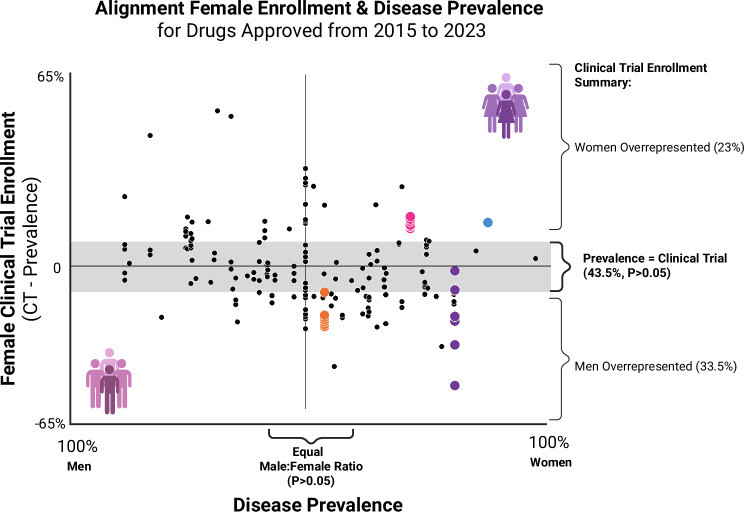
Enrollment percentages of women in 191 clinical trial cohorts supporting drugs approved between 2015 and 2023 (figure excludes sex-specific indications). The X-axis shows the prevalence of each disease indication among men and women (full list in Supplementary Data 4). The Y-axis shows the female clinical trial enrollment for each drug therapy (percentage of females in a clinical trial cohort minus the relative prevalence of the disease indication among females). The gray region represents the range in which observed enrollment does not differ significantly from expected prevalence (43.5% (*n* = 83) of all clinical trials, determined by a two-sided chi-squared test with Benjamini–Hochberg adjustment, adjusted *P*  >  0.05) and does not represent an error band. Women were overrepresented in 23% (*n* = 44) of trials, while women were underrepresented (i.e., men overrepresented) in 33.5% (*n* = 64) of trials. Orange: plaque psoriasis—9 independent clinical trials, female indication prevalence of 54% (i.e., 54% of people affected are women). Pink: migraine—9 independent clinical trials, female indication prevalence of 68.5%. Purple: urinary tract infection—8 independent clinical trials, female indication prevalence of 76%. Blue: osteoporosis—2 independent clinical trials, female indication prevalence of 82%; both trials enrolled only women. Created in BioRender. Zaaijer, S. (2026) https://BioRender.com/. k5d85o8. Source data are provided in this paper.

To address our second question, we tested whether women’s enrollment in late-stage (pivotal) trials reflected their disease burden at the indication level. Using sex-disaggregated enrollment data from US FDA DTSP reports, we compared each trial cohort’s sex ratio with our indication-specific relative prevalence ratio. For this analysis, we excluded the 27 sex-specific indications, leaving 191 trial cohorts. Among these cohorts, female inclusion did not differ from the estimated sex-based prevalence of the targeted indication in 43.5% (*n* = 83) of cases (χ² test, Benjamini-Hochberg adjusted *P* > 0.05; Fig. 4, gray area). Women were overrepresented in 23% (*n* = 44) of cases, and men in 33.5% (*n* = 64). All data is summarized in Supplementary Data 4.

For some indications with multiple treatment approvals, sex-based patterns in trial enrollment diverged from those in population-wide disease burden in notable ways. Across eight trials for therapies of complicated urinary tract infection (UTI; mean relative female prevalence: 76%), women comprised a mean of 50% of trial participants (range: 26–74%), with seven trials under-enrolling women (Fig. 4, purple dots). All nine trials for migraine treatments (mean relative female prevalence: 68.5%) over-enrolled women (range: 84–89%; Fig. 4, pink dots). For osteoporosis treatments (relative female prevalence: 82%), both trials enrolled only women (100%), excluding men (Fig. 4, blue dots).

### Differences over the years

No statistically detectable trend in the enrollment of women in clinical trials was observed over the nine years analyzed (linear regression, F₁,₇ = 0.0142, *P* = 0.909; Supplementary Fig. 1). However, given the limited number of annual observations, this analysis is underpowered and should not be interpreted as evidence of stability over time.

### Biases in female representation for specific therapeutic areas

**Fig. 5:**
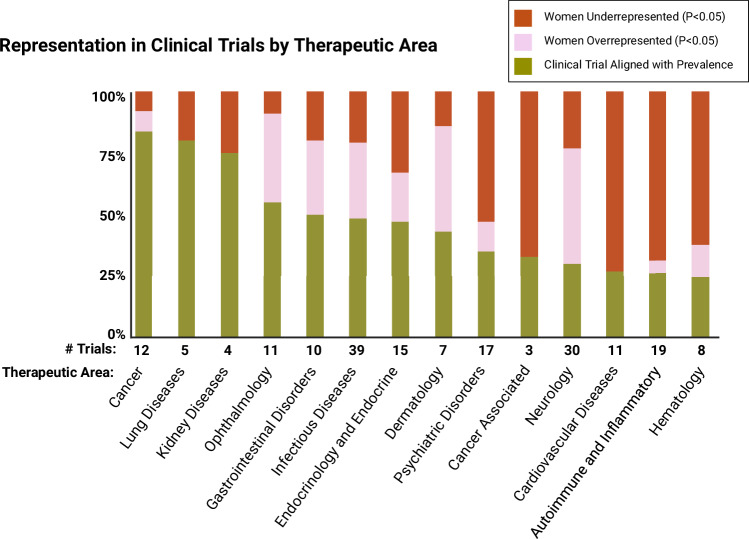
Enrollment percentages of women in clinical trials for specific therapeutic areas, excluding sex-specific indications. The X-axis shows the therapeutic areas by which individual disease indications were binned (full list in Supplementary Data 5). The Y-axis shows the percentage of trials within each therapeutic area for which female enrollment is aligned with expectations based on disease prevalence (green), or for which women are over-enrolled (pink) or under-enrolled (red). Differences in proportions across therapeutic areas were assessed using a two-sided chi-squared test. Exact *P*-values and associated statistics are provided in Supplementary Data 4. Source data are provided in this paper.

We analyzed sex-based enrollment patterns by therapeutic area (Fig. 5) to assess if biases existed for certain disease types. For *Cancer* indications, seventeen trials evaluated treatments for sex-specific cancers (prostate, breast, ovarian, cervical), where enrollment inherently mirrors disease prevalence. Among the 12 trials that tested treatments for non-sex-specific cancers, 83% (*n* = 10) showed enrollment patterns that were aligned with disease prevalence, one over-enrolled women, and the other over-enrolled men.

In stark contrast, among trials for treatments targeting *Cardiovascular Diseases* and *Autoimmune and Inflammatory Diseases* 73% (8 of 11) and 68% (13 of 19) of trials, respectively, underrepresented women. For the latter therapeutic area, underrepresentation of women was driven by trials for plaque psoriasis therapies (8 of 19). This disease has a relatively equal prevalence among the sexes (relative female prevalence: 53.8%); however, female enrollment in trials for drugs targeting plaque psoriasis was on average 30.2% (range: 28–42%). Within this same therapeutic area, rheumatoid arthritis—where women account for ~ 71% of cases—showed high female trial participation: across three trials, mean female enrollment was 80% (range, 79–82%). Women were also represented well in clinical trials that tested lupus nephritis and systemic lupus erythematosus (SLE) therapies (Supplementary Data 4 and Supplementary Data 5).

Across 30 trials for treatments in the area of *Neurology*, 77% aligned with or exceeded the relative female prevalence of indications (9 matched prevalence patterns, while 14 over-enrolled women). This area showed the highest overrepresentation of women in pivotal trials among all therapeutic areas (Fig. 5). Across 17 trials for therapies targeting *Psychiatric Disorders*, 47% aligned with prevalence patterns or over-enrolled women (six matched; two over-enrolled). The majority of drug approvals in this area centered on schizophrenia (five trials), with additional drug development programs focusing on major depressive disorder (MDD; three trials). For MDD (relative female prevalence: 62.4%), two trials matched the relative disease burden among women (female enrollment of 64.7% and 69%, respectively; Supplementary Data 4 and Supplementary Data 5).

## Discussion

### Focus of drug-development portfolios: 1.5-5.5x greater emphasis on female-predominant indications

Our results suggest that, since 1993, the biopharmaceutical industry has become increasingly attentive to disease areas with a higher female burden, as reflected in both approval patterns and the representation of women in pivotal clinical trials. These observations indicate that conditions affecting women receive substantial therapeutic attention within the current drug development landscape. However, progress appears to have plateaued over the past decade, and important gaps remain.

We wondered if absolute case counts—and the market opportunity they imply—might form the primary driver for investment. For example, the female-predominant indication breast cancer accounts for an estimated 310,720 invasive cases plus 56,500 ductal carcinoma in situ (DCIS) cases annually among women, leading to 42,250 deaths; male breast cancer adds a further 2790 cases and 530 deaths^10^. The andrological indication prostate cancer, on the other hand, causes 299,010 new cases and about 35,250 deaths annually^11^. Total case counts of each of these indications are in similar ranges—yet, over the 2015–2023 period, ~ 11 new treatments were approved for breast cancer versus ~ 4 for prostate cancer. Although causality cannot be inferred, it is noteworthy that, in 2023, research investment by the US NIH’s National Cancer Institute (NCI) was roughly 2 × higher for breast cancer than for prostate cancer ($542 M vs. $258 M, respectively)^12^. This gap used to be even higher in relative terms: in 1996, research funding was ~ 4.5 × higher for breast cancer than for prostate cancer ($317 M vs. $71.7 M, respectively)^13^. This funding profile may have enabled the discovery of more and better biomarkers for breast cancer, in turn priming novel therapeutic ideation and successful development of new treatments.

Encouragingly, NCI funding for research on ovarian cancer has risen ~ 3.6-fold since 1996 (from $36.5 M then to $132 M in 2023)^12,13^, paralleling the increase in research funding for prostate cancer. If sustained, this trajectory should catalyze biomarker discovery and, ultimately, lead to new therapeutics.

### Enrollment proportionality: representation of women met or exceeded disease prevalence in 67% of trials

Our second research question assessed whether women are proportionally represented in late-stage clinical development relative to their disease burden. Overall, the findings indicate partial alignment: in 67% of pivotal trials, women’s enrollment met or exceeded indication-specific relative prevalence among females, including 23% (*n* = 44) with overrepresentation. This overrepresentation represents the opposite extreme; for example, in two osteoporosis trials, women comprised 100% of participants, despite men accounting for approximately 18% of osteoporosis cases. At the same time, women are underrepresented in 33% of trials, with these disparities concentrated in specific disease areas (discussed below).

These results should be interpreted within important constraints. First, alignment to disease prevalence serves as a pragmatic benchmark rather than a definitive standard of equitable representation, as prevalence estimates are themselves shaped by patterns of diagnosis and healthcare access. In contexts where women have higher healthcare utilization, observed prevalence may be inflated, and apparent alignment may therefore reflect concordance with an elevated benchmark rather than true proportional inclusion. Conversely, in conditions where diagnostic criteria are historically calibrated to male presentations, prevalence in women may be underestimated; in such cases, alignment may reflect concordance with a lower bound of the true disease burden.

Second, numerical representation alone does not ensure that sex-specific physiology is adequately captured in trial design, analysis, or reporting, and therefore may not translate into the identification of female-specific efficacy or safety outcomes. Third, these findings rely on aggregate comparisons based on binary biological sex and may obscure disparities in participation across racial, ethnic, and socioeconomic subgroups of women.

Taken together, these considerations indicate that alignment with prevalence should not be interpreted as a definitive endpoint of equitable representation, but rather as an informative, context-dependent signal. Within these constraints, our fine-grained, indication-level analyses across therapeutic areas provide a basis to distinguish whether observed underrepresentation reflects structural barriers to enrollment or underlying scientific and clinical complexities that must be addressed to achieve more equitable representation.

### Oncology as an exemplar: clear diagnostics and endpoints support proportionate representation

The therapeutic area of *Cancer* (oncology) illustrates how structural and biological factors can align to support equitable representation of the sexes in clinical trials. Among the 12 trials for treatments of non-sex-specific cancers in our analysis, 10 showed female enrollment patterns consistent with sex-based prevalence ratios in the US. Notably, this contrasts with earlier systematic reviews that reported underrepresentation of women in oncology trials^5–7,14^; this discrepancy is addressed below in the section “Sponsorship and Regulatory Oversight.” Here, we discuss factors that distinguish oncology from other therapeutic areas and that possibly contributed to effective trial enrollment of women in this area.

The first factor is diagnostic precision. Oncology pioneered precision medicine: as sequencing costs fell, and comprehensive tumor profiling became routine, particular types of cancer could be partitioned into molecularly defined subtypes with actionable biomarkers that double as therapeutic discovery guides and trial eligibility criteria. This biomarker-to-therapy mapping reduces diagnostic ambiguity, aligns enrollment with biology, and concentrates treatment effects. Numerous examples illustrate this point: in lung cancer, mutations in *HER1* (encoding the epidermal growth factor receptor [EGFR]) strongly predict benefit from the drug gefitinib^15^. In breast cancer, treatment with alpelisib + fulvestrant prolongs disease progression-free survival of patients with four highly specific tumor cell characteristics: (1) mutations in the metabolic enzyme-encoding gene *PIK3CA*, (2) expression of estrogen and progesterone hormone receptors (HR-positive), (3) no or low expression of human epidermal growth factor receptor 2 (HER2-negative), and (4) previous experience with endocrine therapy^16^ – indicating the level of precision reached. Furthermore, poly (ADP-ribose) polymerase (PARP) inhibitors demonstrate efficacy in patients deficient in BRCA DNA repair proteins^17,18^.

The second factor that distinguishes oncology from other fields is the ability to use objective endpoints. Oncology typically relies on standardized, hard outcomes—overall survival (OS), progression-free survival (PFS), and objective response rate (ORR)—that reduce subjectivity relative to symptom-based scales and thereby limit sex-related variance in assessing disease progression and treatment effects^19^. Although measurement of circulating tumor DNA (ctDNA) is not an FDA-accepted primary efficacy endpoint or validated surrogate, it is increasingly used for patient selection and stratification, minimal residual disease (MRD)-based enrichment to identify high-risk patients, and assessment of early-phase responses to treatment (signal-finding, dose optimization) as supportive evidence alongside clinical endpoints^20^. These practices have advanced the field even more towards objective decision-making.

The third and final factor is the typically greater patient age at the onset of oncological diseases. The median age at cancer diagnosis is ~ 67 years ( ~ 63 years for breast cancer)^21^. Although the age distribution at diagnosis can show a wide spread, with a meaningful proportion of younger patients, the majority of cancer cases still occur at older ages. Consequently, safety restrictions related to patients of reproductive age (e.g., pregnancy exclusions, contraception requirements, and teratogenic risks) are generally less limiting for conducting drug trials in oncology. For example, trials for breast cancer therapies can recruit participants among post-menopausal women, the group that carries the largest burden of this disease^21^.

Together, these factors likely contribute to the relatively balanced representation of the sexes observed in oncology trials when compared to trials in other therapeutic areas.

### Mitigation of reproductive risks: preclinical optimization to enable inclusive enrollment

Trials for therapies that target *Autoimmune Diseases*, especially psoriasis, show a pattern opposite to the one that can be observed for oncology trials. Although the relative female prevalence of psoriasis is 54%, all nine pivotal trials focusing on this disease indication underrepresented women, with a mean female enrollment of only 31.6% (range: 29–42.5%).

Psoriasis manifests in both early- and late-onset forms, but the early-onset subtype—by far the most common one—generally occurs at ages 16–22 in women and 30–39 in men^22–24^. Coincidentally, the onset of this disease subtype in women overlaps with reproductive years. This could disproportionately limit female eligibility for trial participation through pregnancy and lactation exclusions, contraception requirements, and additional safety-monitoring obligations^25^.

While the NIH Revitalization Act aimed to address concerns over reproductive safety with contemporary frameworks that included protocols to mitigate risks for the teratogenic potential of new drugs, the examples of drugs that do still pose reproductive safety risks are extensive, specifically among autoimmune therapies. For instance, the drugs methotrexate (a dihydrofolate reductase inhibitor) and acitretin (a retinoid receptor agonist) are established teratogens with significant fetal risks^25^. Current FDA guidelines on clinical trial enrollment practices^26^ require at least one embryo-fetal development (EFD) study before women of childbearing potential can be included. Although valuable for hazard identification, risks identified in EFD studies—usually conducted on animals—translate imperfectly to humans, with a concordance of only 60–70% and a high false-positive rate^27–29^. In practice, this uncertainty has likely caused a continuation of conservative practices to exclude women from trials, either through choices made by trial sponsors or by female candidate enrollees themselves. Such choices may explain our observation that women are systematically underrepresented in trials for psoriasis treatments.

Emerging human-based preclinical models offer possible solutions. Stem cell-derived assays, such as the devTOX quickPredict™ platform that uses biomarker profiles in human induced pluripotent stem cells (hIPSCs), have achieved >80% predictive accuracy across more than 1000 compounds, outperforming many animal-based EFD studies^30–33^. The FDA is currently considering stem cell-derived assays under a weight-of-evidence framework^28^. Once validated and incorporated into regulatory frameworks, these models could lessen reliance on animal studies and, crucially, strengthen preclinical safety assurances—enabling broader inclusion of reproductive-age women in trials for autoimmune therapies.

### Endpoint complexity

Diagnostic challenges and difficulties with identifying objective endpoint metrics may form further foundational inhibitions for improving representation of women in clinical trial cohorts. Oncology offers a template in which the use of standardized endpoints (OS, PFS, ORR) constrains subjectivity^19^. *Autoimmune Disease* indications occupy an intermediate position; endpoint metrics frequently consist of composite indices that mix laboratory measures with clinician- and patient-reported components. For example, the American College of Rheumatology 20%, 50% or 70% improvement index (ACR20/50/70) combines quantitative measurements of markers for inflammation, such as C-reactive protein (CRP), with a Health Assessment Questionnaire (HAQ). Clinical studies that use such indices may need to reckon with more variance in resulting datasets relative to studies where clinical outcomes can be measured following objective metrics alone^34^.

Studies in the areas of *Neurology* and *Psychiatric Disorders* rely even more on perception-sensitive outcomes. Trials for migraine therapies overrepresented women (9/9 clinical trials, range of female enrollment: 84–89%), as did one for pain treatment (79%). Many migraine studies use endpoints such as “2 h pain freedom” or “most bothersome symptom (MBS) freedom”, while psychiatric trials frequently depend on the Hamilton Depression Rating Scale (HAM-D) and other scales; such subjective endpoints are vulnerable to recall bias, inter-rater variability, and high placebo response, which can obscure true treatment effects^35–38^. These concerns are amplified for women in some cases: self-reported endpoints for treatments of “menstrual migraine”, for example, have been proven to show poor accuracy^39,40^. The use of subjective endpoints can lead to heterogeneous case definitions and may have discouraged enrollment of female trial participants who displayed symptoms linked to the menstrual cycle that may overlap with migraine-associated symptoms. Increasingly, the solution has been to make adjustments to trial design rather than excluding women from trials. This has been aided by the FDA’s enrichment guidance (finalized on March 15, 2019)^41^, which endorses prospective selection of event-rate subgroups and pre-specification of heterogeneity analyses. For example, migraine-related trials can use a specific peri-menstrual window ( − 2 to + 3 days from menses onset) plus prospective time-locked e-diaries to remove the need to recall data^42^. These structured elements reduce misclassification, stabilize endpoint variance, and improve signal-to-noise ratios. This enables inclusion rather than avoidance of subgroups of women. However, our observed overrepresentation of women in these trials does result in an underpowered detectability of treatment efficacy and safety signals among males^43^. Trial sponsors’ choices for this enrollment pattern may either indicate that variance among women is still magnitudes higher than among men or that there may be challenges with cross-utilizing the same protocols for both female and male participants.

### Diagnostic complexity

Sex-divergent symptom profiles create barriers to diagnosis and equitable enrollment. When clinical presentation differs by sex, case definitions and screening tools calibrated to one profile can miss the other, and eligibility criteria keyed to those features can propagate underrepresentation of one of the sexes. For example, men with schizophrenia more often exhibit negative symptoms (e.g., social withdrawal, blunted affect), whereas women more often present with positive symptoms (e.g., hallucinations, delusions)^44–47^. These differences can influence who is diagnosed, referred, and ultimately enrolled in trials, biasing both prevalence estimates and trial cohort compositions.

Cardiovascular medicine offers a parallel case. Women more often present with non-chest-pain symptom clusters and are underdiagnosed when male-derived thresholds are used (e.g., non-sex-specific troponin cut-offs)^48–52^. Accordingly, the female under-enrollment observed for 8 of 11 trials in our dataset (also noted by Scott et al.^53^ and others) likely reflects bias in upstream diagnostics rather than site-level recruitment. The stakes are substantial**:** McKinsey estimates that closing women’s heart-health gaps could prevent losses of ~ $28 billion to the US economy^54^.

These examples, and more, highlight that diagnostic issues cannot be solved during the design phase of clinical trials. Rather, solutions need to be sought more systemically up and down the drug discovery pipeline, across phases and organizational departments:

First, molecular diagnostics that provide objective measures of disease activity and treatment response can promote more equitable enrollment. For instance, recent sex-informed transcriptomic and epigenomic profiling of schizophrenia patients described greater synaptic-signaling disruption in men and stronger immune-metabolic alterations in women^55,56^, offering a basis for the development of biomarker panels and stratifiers to guide trial eligibility, cohort stratification, and treatment response evaluation. Applied prospectively, such tools can sharpen disease definitions, improve prevalence estimates (and market sizing), reduce endpoint variance, and yield clearer therapeutic effect estimates—thereby reducing incentives to default to single-sex enrollment.

Second, divergent symptom presentation and physiology should be studied to a greater extent, including in the pre-clinical drug discovery phase. Artificial intelligence (AI)-enabled tools offer potential solutions for broadening the number of objective diagnostics and addressing sex-divergent phenotypes. In *Dermatology*, deep learning systems quantify erythema, scaling, and lesion distribution from images, reducing inter-observer variability in assessing disease symptoms across patients with varying skin tones and of different sexes^57^. In *Neurology*, smartphone diaries and wearable-based models can capture migraine-induced headache frequency and intensity with greater precision than human observers can, decreasing sex-related differences in reporting^35,58,59^. In *Psychiatric Disorders*, digital phenotyping and speech-analysis algorithms increasingly detect subtle symptom clusters that differ between men and women but are overlooked by traditional metrics. In *Cardiovascular Diseases*, AI-enhanced electrocardiogram (ECG) models can now identify sex-dependent repolarization patterns, with “sex discordance scores” predicting excess risk of cardiovascular death and heart failure in women^60,61^. Routine capture of menopausal status, contraceptive use, and—where relevant—menstrual cycle phase in women can further reduce unexplained variance. Pre-specifying sex-by-treatment interactions and powering secondary analyses accordingly preserves overall trial efficiency while producing interpretable readouts across different subgroups of patients.

Overall, female enrollment matched or exceeded indication prevalence in 67% of pivotal trials—most often in therapeutic areas that are able to benefit from the availability of precise diagnostics, objective endpoints, and older participant cohorts (e.g., *Cancer*). For the remaining 33% of trials, which underrepresented women, one or more of these conditions were generally absent. This observation suggests that female enrollment in clinical trials may continue to plateau if certain structural fixes are not implemented. These should include: (i) use of validated, human cell-based teratogenic risk assays of higher accuracy to enable inclusion of women of reproductive potential; (ii) optimization of endpoints for conditions that are currently assessed using subjective metrics (e.g., tracking migraine using prospective e-diaries and menstrual cycle-anchored measures); and (iii) diagnostic upgrades, including use of genomics or AI-supported tools that capture sex-differentiated signals.

It remains unclear whether enrollment merely reflects disease prevalence—except where technical or biological constraints apply—or whether sites actively manage sex ratios to meet sponsor targets.

### Sponsorship and regulatory oversight

Beyond disease biology and trial design, sponsor type does appear to exert a measurable influence on female representation in clinical trial cohorts. Our dataset, which only includes data from industry-sponsored pivotal trials that led to FDA-approved drugs, revealed an average female enrollment of 54.5% (50.8% when excluding trials focused on sex-specific indications). This is higher than prior estimates based on data from ClinicalTrials.gov, which placed it at 38–49%^2–7^. Independent analyses corroborate the existence of this difference: between 2016 and 2019, industry-sponsored phase III trials enrolled women at higher rates (38.1%) than government-funded trials (29.2%), with the largest disparity observed in trials for treatments of *Psychiatric Disorders* (54.3% vs. 33.5%, respectively)^2^. Similar patterns have been noted for oncological and cardiovascular disease trials^3,7,14^.

Regulatory scrutiny and accountability likely contribute to this difference. Industry trials designed for approval are subject to FDA oversight and investor expectations, creating stronger incentives to ensure balanced enrollment. In contrast, government-funded studies often involve exploratory science, non-interventional or early-phase insights into drugs’ mechanisms-of-action, where representational equity may be a lower priority. This divergence highlights how trial sponsorship structure can shape patient demographics in clinical studies.

### Female inclusion and trial success

Our findings raise the hypothesis that higher female inclusion may itself contribute to regulatory approval success. The FDA DTSP reports only summarize studies that led to approved products. This is unlike the ClinicalTrials.gov database, which also includes ongoing, withdrawn, and failed studies. Consequently, the higher relative female enrollment we observe may reflect a survivorship effect: trials that succeed tend to be those with more balanced inclusion of each sex.

Token female inclusion may create the appearance of a broadly representative trial cohort. If responses differ by sex, under-enrolling women leaves the female subgroup underpowered and may miss beneficial effects. In addition, when data from such a cohort is pooled across the sexes, the signal among the male participants must be strong enough to overcome the increased variance in the data caused by the inclusion of a small group of female participants. Therefore, underrepresenting women in trials could heighten the risk of false-negative trial outcomes.

Moreover, trials with poorly representative enrollment can prompt FDA requests for confirmatory or bridging studies, potentially resulting in costly delays to drug approval.

Future work linking sex-specific enrollment patterns in unsuccessful, withdrawn trials to clinical outcomes will be essential to determine whether stronger female inclusion causally increases the likelihood of drug approval.

### Limitations

Several limitations of our analyses warrant discussion. First, our estimates of sex-specific disease burden draw on both prevalence and incidence data, which capture different aspects of disease epidemiology. Chronic conditions are typically measured using prevalence, reflecting the population currently living with disease, whereas acute conditions are better captured by incidence, reflecting new cases over time^62^. Because these metrics are not directly comparable, our analyses rely on sex ratios to mitigate potential biases when benchmarking trial enrollment against disease burden. Nevertheless, different denominators address different normative questions and may produce different perspectives on representation.

Second, epidemiological estimates themselves may be influenced by healthcare utilization patterns and diagnostic frameworks. Women generally seek healthcare more frequently than men^63^, which may inflate prevalence estimates in some contexts. Conversely, diagnostic criteria historically calibrated on male phenotypes can underrecognize disease in women, as documented for *Cardiovascular Diseases*^64^ and several chronic conditions^52,64–66^. As a result, epidemiological benchmarks—such as those derived from SEER and related datasets—should not be interpreted as definitive measures of equity. Instead, we use them as pragmatic reference points anchored in the same epidemiological data that commonly inform clinical trial design and disease burden estimates in biomedical research.

Third, disease burden estimates vary across populations and geographic regions. For example, in sub-Saharan Africa 63% of new HIV infections are found among women, whereas in the US women represent 19% of new cases^67,68^. In addition, in the US 82% of newly diagnosed osteoporosis patients are women, but this falls to 62% when diagnoses are assessed globally^69^. To minimize this variability, we prioritized US-based prevalence and incidence estimates where available. Nevertheless, regional differences in disease epidemiology may limit the generalizability of some comparisons.

Fourth, our analysis relies on the level of disease indication used in regulatory approvals, which can mask heterogeneity within molecularly defined subtypes. As precision medicine advances, disease categories are increasingly subdivided by genetic or biomarker-defined characteristics that may show distinct sex distributions (e.g., the 2021 approval of amivantamab [Rybrevant] for locally advanced or metastatic non–small cell lung cancer [NSCLC] with EGFR exon 20 insertion mutations). NSCLC accounts for about 85% of lung cancers. However, specific molecular subtypes within NSCLC may exhibit different sex distributions. In a recent multinational study of patients with resected stage IA–IIIB NSCLC, EGFR mutations were present in 51.0% of patients overall, and women had a higher EGFR mutation rate than men (64.0% vs. 36.4%)^70,71^. These findings illustrate how molecularly defined subtypes can differ in epidemiology and patient characteristics compared with the broader disease category. As clinical trials become increasingly biomarker-specific, incorporating finer molecular classifications in future analyses may provide additional insight into sex-specific disease distributions and help refine representation targets in clinical trials.

Finally, our study evaluates representation using the binary sex categories reported in the FDA DTSP data. These regulatory datasets capture sex as recorded in trial documentation and do not include gender identity or consistent intersectional breakdowns (e.g., sex-by-race or socioeconomic status). Consequently, representation of gender-diverse individuals and subgroup disparities in clinical trial cohorts cannot be assessed within this framework, and improvements in overall female participation may mask differences across demographic groups. Our findings should therefore be interpreted as reflecting patterns in representation at the aggregate sex level rather than a comprehensive assessment of gender equity.

More than three decades after the NIH Revitalization Act was signed into law, our analysis shows that the biopharmaceutical industry has become attentive to women’s health. Despite the remaining backlog of unmet needs in women’s health, this shift is evident in: (i) relatively large, targeted investments leading to approved treatments for indications with a high female burden, and (ii) greater attention to trial enrollment according to disease burden. Nonetheless, despite these gains, female enrollment in clinical trials appears to have plateaued. Sustained progress will require advances in diagnostics, increasing the number of diseases for which objective endpoints can be leveraged, and improving tools to assess risks specific to women of reproductive potential. Addressing these gaps will be critical to further reduce sex biases in clinical trials.

## Methods

### Human subjects statement

This study uses publicly available, aggregated data and does not involve individual-level human participant data. Institutional review board approval and informed consent were not required.

All analyses were conducted using aggregated, cohort-level data as reported in the source datasets.

### Data curation methodology

#### Regulatory data framework

The analysis incorporated New Molecular Entities (NMEs) and Biologics License Applications (BLAs) from US FDA DTSP reports (Fig. 1). All demographic and trial data were captured as documented during the initial regulatory review period, reflecting snapshots at original approval without post-marketing updates.

#### Demographic data handling

Sex classifications were derived from sponsor-submitted trial participant data.

#### Data harmonization protocol

*Pharmaceutical Compound Annotation*

- Brand/generic drug names were standardized using annual approval summaries from *Nature Reviews Drug Discovery* (2015, *n* = 45^72^, 2016, *n* = 22^73^, 2017, *n* = 46^74^; 2018, *n* = 59^75^; 2019, *n* = 48^76^; 2020, *n* = 53^77^; 2021, *n* = 50^78^; 2022, *n* = 37^79^; 2023, *n* = 55^80^).
- For some drugs, two independent drug trials were listed (*n* = 8), while several other drugs were listed with two disease indications each (*n* = 13). In these cases, we augmented so that one drug is associated with one trial for one indication.

### Therapeutic area assignment

FDA DTSP records do not provide a consistent therapeutic area classification across years. While disease annotations are available for a subset of approvals, these are not systematically reported and do not follow a stable controlled vocabulary. To enable cross-approval comparisons, we implemented a structured workflow to assign each approval to a single standardized therapeutic area.

For each approval, we extracted the drug name and FDA-approved indication text (disease/condition text). FDA-provided disease annotations, when available, were not used as inputs for classification but were used to construct and validate the classification framework. The objective was to map heterogeneous indication text to a consistent set of therapeutic area categories.

#### Construction of therapeutic area categories

Therapeutic area categories were derived by reviewing FDA disease annotations where available and consolidating recurring disease domains into a study-specific classification schema. This schema defined a fixed set of allowable labels used for all subsequent classification.

#### Large language model (LLM)-assisted classification

Therapeutic area assignment was performed using Perplexity AI. For each approval, the model was provided with the drug name and indication text and instructed to assign exactly one therapeutic area from the predefined category list.

This task was formulated as a single-label classification problem with a fixed (closed) set of categories, meaning that each approval was assigned to exactly one category from a predefined list of therapeutic areas. The model was restricted to selecting from this list and was not permitted to generate new or free-text categories, ensuring consistent and comparable outputs across approvals. Prompts were applied uniformly using identical instructions.

Perplexity AI was selected due to its search-integrated architecture, which combines language modeling with retrieval of external information, supporting grounding in established clinical terminology. In contrast, general-purpose LLMs such as Generative Pre-trained Transformer (GPT)-based systems are optimized for reasoning, abstraction, and handling ambiguous inputs, but may generate explanatory text or additional interpretation unless strictly constrained. For the present task, which required assignment to a fixed category set, concise and constraint-adherent outputs were preferred. Perplexity AI, therefore, proved well-suited to this application, generally producing fact-oriented responses with minimal extraneous text. The classification framework itself is model-agnostic and could be implemented using other LLMs under similar constraints.

The following is an example of how the classification framework was applied in Perplexity AI to categorize one of the drugs in our study:

“You are given a drug approval and its FDA-approved indication. Assign the approval to exactly one therapeutic area from the following list: Autoimmune and Inflammatory Diseases; Cancer; Cancer Associated; Cardiovascular Diseases; Dermatology; Endocrinology and Endocrine Disorders; Gastrointestinal Disorders; Hematology; Infectious Diseases; Kidney Diseases; Lung Diseases; Neurology; Ophthalmology; Psychiatric Disorders; Reproductive Health.

Rules:

- Return only one category from the list.
- Do not create new categories.
- Use the indication text as the primary basis for classification.

Drug: Pembrolizumab

Indication: Metastatic non-small cell lung cancer"

Expected output (LLM response): Cancer.

This example illustrates the constrained classification task in which the model maps indication text to a predefined therapeutic area category without generating additional explanatory text.

#### Harmonization and category consolidation

Following initial classification, predefined rules were applied to ensure consistency and reduce sparsity. Pain-related indications were grouped under *Psychiatric Disorders* (e.g., opioid withdrawal and acute pain therapies such as Lucemyra and Olinvyk), while sleep disorders and hypoactive sexual desire disorder were grouped under *Neurology* (e.g., insomnia therapies such as Dayvigo and Quviviq). Indications related to complications of cancer treatment (e.g., chemotherapy-induced nausea and vomiting, chemotherapy-induced myelosuppression) were assigned to a distinct category, *Cancer Associated*. Categories with very low frequency (*n* ≤ 2 approvals) were merged into clinically related groups.

#### Validation against FDA annotations

To assess classification reliability, we performed a validation analysis using approvals with FDA-provided disease annotations (*n* = 129). These annotations were mapped to the same category schema using study-defined category definitions and compared with LLM-derived assignments.

Concordance was defined as exact agreement at the individual approval level. Agreement was observed in 125 of 129 approvals (96.9%), corresponding to a discordance rate of 3.1% (4/129).

#### Human review

For approvals lacking FDA disease annotations (*n* = 85), as well as for discordant cases, each author independently reviewed the LLM-derived assignments for appropriateness relative to the indication text and consistency with the predefined category schema and harmonization rules. Discrepancies were resolved by consensus. This step served as quality control rather than primary classification.

#### Final dataset and exclusions

Following classification, harmonization, and review, each approval was assigned to exactly one therapeutic area. A total of 214 approvals were annotated using this workflow. Final assignments are reported in Supplementary Data 2 alongside the original indication text.

Orphan drugs were excluded from downstream analyses due to limitations in linking disease burden metrics to enrollment patterns. Approvals categorized as imaging therapies were also excluded, as they were not suitable for analyses of clinical trial enrollment. Three additional indications were excluded due to a lack of reliable prevalence or incidence data.

### Inclusion of specific disease annotations and prevalence or incidence statistics

We estimated sex-specific disease burden via structured searches prioritizing US data where possible (CDC/SEER – Supplementary Data 1). For each indication, we chose the epidemiologically appropriate denominator—prevalence for chronic/relapsing conditions, incidence for acute diseases—selecting the most conservative estimate when sources conflicted. We further assessed study quality (sample size, case definition, bias, timeframe, representativeness). For example, for Alzheimer’s disease, we benchmarked enrollment to sex-disaggregated prevalence (not incidence)^81^. Because women live longer, their share of prevalence exceeds that of incidence. Prevalence, therefore, better reflects the population living with this disease—i.e., people eligible for trials and with the longest exposure to potential benefits and risks of a new drug.

All values were harmonized to %W | D (percent women among people affected); we use the term “prevalence” as shorthand and note when incidence is used.

### Computation of prevalence estimates

For each indication, we computed the probability for a random woman vs. a random man in the US to have the disease based on the sex-specific disease burden and the percentage of females and males in the US population according to US Census data (50.5% and 49.5%, respectively). For example, if the ratio of women vs. men among affected cases is equal (a 1:1 ratio), then the chance that someone burdened by the disease is a woman or a man equals the frequency of women compared to men in the US Census population: a 50.5% probability that the person is a woman vs. 49.5% that the person is a man. Following this logic, it was possible to compute a relative prevalence estimate for each disease indication among women in the US population using Bayes’ theorem with demographic and disease prevalence data:

Let:

- $P({D|W})$: be the prevalence of a disease in women
- $P({D|M})$: be the prevalence of a disease in men
- $P(W):$ be the prevalence of women in the population according to the US Census (50.5%)
- $P(M):$ be the prevalence of men in the population $(=1-P(W))$

The total probability that people afflicted by the disease in the US ($P(D)$) as females or males will then be:

1 $$P\left(D\right)=P\left(D | W\right)\cdot P\left(W\right)+P\left(D | M\right)\cdot P(M)$$

The probability that a person who has the disease in the US is a woman ($P({W|D})$) can then be expressed as:

2 $$P\left(W | D\right)=\frac{P\left(D | W\right)\,\cdot P(W)}{P(D)}$$

### Statistical analysis

For each trial in our panel, we computed if representation of women and men in that clinical trial was representative for the prevalence among the sexes of the disease indication targeted by the treatment, as determined by chi-squared testing. The two-sided *P*-values from the chi-squared tests were then subjected to the Benjamini-Hochberg procedure to calculate adjusted *P*-values at a False Discovery Rate (FDR) of 5%^82^. A test outcome was deemed significant when the adjusted *P*-value reached below a threshold of 0.05 (adjusted *P* < 0.05). A significant outcome indicated that the male/female ratio in a trial was not representative of a disease indication’s prevalence among the sexes in the US population.

### Reporting summary

Further information on research design is available in the Nature Portfolio Reporting Summary linked to this article.

## Supplementary information

Supplementary Information

Description Of Additional Supplementary File

Supplementary Data 1

Supplementary Data 2

Supplementary Data 3

Supplementary Data 4

Supplementary Data 5

Reporting summary

Transparent Peer Review file

## Source data

Source data

## Data Availability

The source data underlying Figs. 2–5 and Supplementary Fig. 1 are provided with this paper. Source data are provided in this paper.
