# Supplementary material for: Sex representation in trials relative to indication-specific disease burden in FDA-approved drugs (2015–2023)
Source: Nat Commun. 2026 Jun 23;17:6962. doi: 10.1038/s41467-026-74469-z (PMC13392115; doi:10.1038/s41467-026-74469-z)
Supplement: Supplementary file 1 — Supplementary Information [file 41467_2026_74469_MOESM1_ESM.pdf]

Supplementary Figure 1

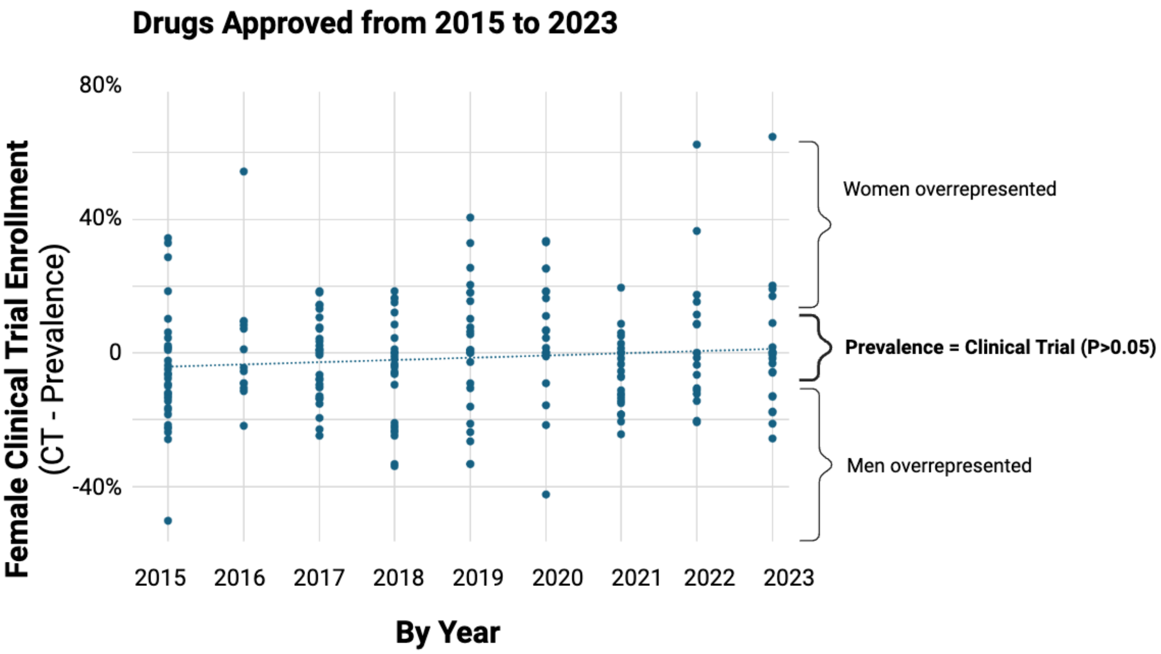

Supplemental Fig. S1 | Female clinical trial enrollment relative to disease prevalence for drugs approved from 2015 to 2023, by year. Each dot represents one pivotal clinical trial cohort (n = 191; sex-specific indications excluded). The Y-axis shows the difference between the percentage of women enrolled in each trial and the sex-based relative prevalence of the corresponding disease indication among women (CT − Prevalence). Values above zero indicate overrepresentation of women; values below zero indicate overrepresentation of men. No statistically detectable trend in the enrollment of women was observed over the nine years analyzed (linear regression, $F_{1,7} = 0.0142$, $P = 0.909$); however, given the limited number of annual observations, this analysis is underpowered. Source data are provided with this paper.
