# Supplementary material for: Sex representation in trials relative to indication-specific disease burden in FDA-approved drugs (2015–2023)
Source: Nat Commun. 2026 Jun 23;17:6962. doi: 10.1038/s41467-026-74469-z (PMC13392115; doi:10.1038/s41467-026-74469-z)
Supplement: Supplementary file 2 — Description Of Additional Supplementary File [file 41467_2026_74469_MOESM2_ESM.pdf]

Description of Additional supplementary files

Supplementary data 1:

Sex Representation in Trials Relative to Indication-Specific Disease Burden in FDA-Approved Drugs (2015–2023)

Supplementary data 2:

Prevalence by disease indication

Supplementary data 3:

Sex-biased prevalence ratios by indication: σ-binned counts and shares

Supplementary data 4:

Trial Counts and Alignment of Women's Enrollment with Prevalence

Supplementary data 5:

Female representation across therapeutic areas
