# Supplementary material for: Sex representation in trials relative to indication-specific disease burden in FDA-approved drugs (2015–2023)
Source: Nat Commun. 2026 Jun 23;17:6962. doi: 10.1038/s41467-026-74469-z (PMC13392115; doi:10.1038/s41467-026-74469-z)
Supplement: Supplementary file 3 — Supplementary Data 1 [file 41467_2026_74469_MOESM3_ESM.pdf]

SUPPLEMENTAL DATA 1

Sex Representation in Trials Relative to Indication-Specific Disease Burden in FDA-Approved Drugs (2015–2023)

Indication	Fraction Women (compared to men)	Citations
ABSSSI - Acute bacterial skin and skin structure infections	0.29	[1]
Acne vulgaris	0.63	[2]
Actinic keratosis	0.55	[3]
Acute uncomplicated influenza	0.53	[4]
ADHD	0.37	[5]
Alopecia areata	0.61	[6]
Alzheimer disease	0.67	[7]
Assess fallopian tube patency in women with known or suspected infertility	1	NA
Asthma (Severe)	0.71	[8]
Atherosclerotic cardiovascular disease ASCVD, [HeFH or]	0.44	[9]
Atopic dermatitis	0.61	[10]
Bacterial vaginosis	1	NA
Basal cell carcinoma	0.34	[11]
Bipolar 1 disorder (and Schizophrenia)	0.5	[12]
Bladder cancer	0.20	[13]
Blood pressure in adults with septic or other distributive shock	0.51	[14]
Breast cancer	0.99	[15]
C. difficile recurrence	0.55	[16]
CABP	0.43	[17]
Cervical cancer	1	NA

**Chemotherapy-induced emesis**	0.74	[18]
**Chemotherapy-induced myelosuppression**	0.38	[19]
**Chronic heart failure**	0.38	[20]
**Chronic idiopathic constipation**	0.71	[21]
**CKD with type 2 diabetes**	0.5	[22]
**Colorectal cancer**	0.43	[23]
**COPD**	0.60	[24]
**COVID-19**	0.30	[25]
**CSCC**	0.38	[26]
**Deep vein thrombosis (and risk of stroke and systemic embolism in NVAF**	0.50	[27]
**Diabetes**	0.42	[28]
**Diabetes, Type 2**	0.48	[29]
**DLBCL and high-grade B-cell lymphoma**	0.41	[30]
**DME, [and nAMD]**	0.60	[31]
**Dry eye disease**	0.66	[32]
**EGFR exon 20-mutated NSCLC**	0.64	[33]
**Endometrial cancer**	1	NA
**Fat below the chin**	0.5	NA
**Female contraception**	1	NA
**Geographic atrophy secondary to AMD**	0.5	[32]
**Glabellar lines**	0.5	NA
**Glabellar lines associated with corrugator and/or procerus muscle activity**	0.5	NA
**Gout**	0.25	[34]
**Growth hormone deficiency**	0.33	[35]
**HCV**	0.30	[36]
**Head lice**	0.5	NA

**Heart failure**	0.38	[20]
**HeFH [or ASCVD]**	0.5	[37]
**Helicobacter pylori infection**	0.47	[38]
**HIV**	0.19	[39]
**Hospital-acquired and ventilator-associated bacterial pneumonia caused by susceptible ABC**	0.312	[40]
**Hot flashes caused by menopause**	1	NA
**Hyperkalaemia**	0.45	[41]
**Hypoactive sexual desire disorder**	0.6667	[42]
**Hypoglycaemia (Severe)**	0.64	[43]
**IBS with constipation**	0.71	[44]
**Impetigo due to Staphylococcus aureus or Streptococcus pyogenes**	0.30	[1]
**Incidence of infection in non-myeloid malignancies, with myelosuppressive drugs**	0.372	[45]
**Insomnia**	0.599	[46]
**Iron deficiency anaemia**	0.70	[47]
**Irritable bowel syndrome**	0.61	[48]
**LDL lowering**	0.43	[49]
**Lupus nephritis**	0.7964	[50]
**Macular degeneration (Wet age-related) (Rein, 2022)**	0.5	[32]
**Major depressive disorder**	0.62	[51]
**Migraine**	0.68	[52]
**Multiple sclerosis**	0.76	[53]
**Myocardial infarction, repeat coronary revascularization, and stent thrombosis**	0.38	[54]
**nAMD [and DME] (Rein, 2022)**	0.5	[32]
**Nausea and vomiting after surgery**	0.62	[55]
**Nausea and vomiting associated with chemotherapy**	0.73	[56]

**Neutropenia**	0.35	[57]
**Open-angle glaucoma or ocular hypertension — reduction of elevated IOP (Kapetanakis, 2016)**	0.43	[58]
**Opioid withdrawal**	0.5	NA
**Opioid-induced constipation**	0.71	[59]
**Osteoporosis**	0.82	[60]
**Overactive bladder**	0.51	[61]
**Pain (Acute)**	0.53	[62]
**Pain associated with endometriosis**	1	NA
**Parkinson disease**	0.31	[63]
**Partial onset seizures (Epilepsy)**	0.59	[64]
**Plaque psoriasis**	0.538	[65]
**Postpartum depression**	1	NA
**Procedural sedation**	0.5	NA
**Prostate cancer**	0	NA
**Pruritus associated with CKD**	0.34	[66]
**Psychosis, Hallucinations and delusions associated with Parkinson disease psychosis**	0.4	[67]
**Renal cell carcinoma**	0.34	[68]
**Reversal of neuromuscular blockade during surgery**	0.58	[69]
**Rheumatoid arthritis**	0.71	[70]
**Risk of stroke and systemic embolism in NVAF, [and deep vein thrombosis]**	0.55	[71]
**RSV lower respiratory tract disease**	0.61	[72]
**Schizophrenia**	0.5	[73]
**Secondary hyperparathyroidism in patients with chronic kidney disease on haemodialysis**	0.53	[74]
**SLE**	0.90	[75]
**Tardive dyskinesia**	0.55	[76]

**Thrombocytopenia**	0.56	[77]
**To prevent pregnancy**	1	NA
**Travellers' diarrhoea**	0.55	[78]
**Ulcerative colitis**	0.61	[48]
**Urinary tract (and complicated intra-abdominal infections)**	0.76	[79]
**Urothelial cancers**	0.23	[13]
**Vulvovaginal candidiasis**	1	NA

1. Castleman, M. J. *et al.* Innate sex bias of staphylococcus aureus skin infection is driven by α-hemolysin. *J. Immunol.* **200**, 657–668 (2018).
2. Collier, C. N. *et al.* The prevalence of acne in adults 20 years and older. *J. Am. Acad. Dermatol.* **58**, 56–59 (2008).
3. Navsaria, L. J. *et al.* Incidence and treatment of actinic keratosis in older adults with medicare coverage. *JAMA Dermatol.* **158**, 1076–1078 (2022).
4. Morgan, R. & Klein, S. L. The intersection of sex and gender in the treatment of influenza. *Curr. Opin. Virol.* **35**, 35–41 (2019).
5. Attention-Deficit/Hyperactivity Disorder (ADHD). *National Institute of Mental Health (NIMH)* https://www.nimh.nih.gov/health/statistics/attention-deficit-hyperactivity-disorder-adhd.
6. Mostaghimi, A. *et al.* Trends in prevalence and incidence of alopecia areata, alopecia totalis, and alopecia universalis among adults and children in a US employer-sponsored insured population. *JAMA Dermatol.* **159**, 411–418 (2023).
7. Alzheimer's Disease Facts and Figures. *Alzheimer's Association* https://www.alz.org/alzheimers-dementia/facts-figures.
8. Lin, R. Y. & Lee, G. B. The gender disparity in adult asthma hospitalizations dynamically relates to age. *J. Asthma* **45**, 931–935 (2008).
9. Kwak, S. *et al.* Machine learning reveals sex-specific associations between cardiovascular risk factors and incident atherosclerotic cardiovascular disease. *Sci. Rep.* **13**, 9364 (2023).
10. Ng, A. & Boersma, P. *Diagnosed Allergic Conditions in Adults: United States, 2021*. http://dx.doi.org/10.15620/cdc:122809 (2023) doi:10.15620/cdc:122809.
11. Wu, S., Han, J., Li, W.-Q., Li, T. & Qureshi, A. A. Basal-cell carcinoma incidence and associated risk factors in U.S. women and men. *Am. J. Epidemiol.* **178**, 890–897 (2013).
12. Bipolar Disorder. *National Institute of Mental Health (NIMH)* https://www.nimh.nih.gov/health/statistics/bipolar-disorder (2017).
13. SEER. Cancer of the Urinary Bladder - Cancer Stat Facts. *SEER* https://seer.cancer.gov/statfacts/html/urinb.html (2023).
14. Pietropaoli, A. P., Glance, L. G., Oakes, D. & Fisher, S. G. Gender differences in mortality in patients with severe sepsis or septic shock. *Gend. Med.* **7**, 422–437 (2010).
15. WHO. Breast cancer. https://www.who.int/news-room/fact-sheets/detail/breast-cancer (2024).

16. Ma, G. K., Brensinger, C. M., Wu, Q. & Lewis, J. D. Increasing incidence of multiply recurrent Clostridium difficile infection in the United States: A cohort study. *Ann. Intern. Med.* **167**, 152–158 (2017).
17. Corica, B., Tartaglia, F., D'Amico, T., Romiti, G. F. & Cangemi, R. Sex and gender differences in community-acquired pneumonia. *Intern. Emerg. Med.* **17**, 1575–1588 (2022).
18. Mosa, A. S. M., Hossain, A. M., Lavoie, B. J. & Yoo, I. Patient-related risk factors for chemotherapy-Induced Nausea and vomiting: A systematic review. *Front. Pharmacol.* **11**, 329 (2020).
19. Epstein, R. S. *et al.* Real-world burden of chemotherapy-induced myelosuppression in patients with small cell lung cancer: a retrospective analysis of electronic medical data from community cancer care providers. *J. Med. Econ.* **25**, 108–118 (2022).
20. Heart disease deaths - Health, United States. https://www.cdc.gov/nchs/hus/topics/heart-disease-deaths.htm (2024).
21. Felicia B, LeClere, Ph,D,, Abigail J, Moss. Prevalence of Major Digestive Disorders and Bowel Symptoms, 1989. Preprint at https://www.cdc.gov/nchs/data/ad/ad212.pdf (1992).
22. Wu, B. *et al.* Understanding CKD among patients with T2DM: prevalence, temporal trends, and treatment patterns-NHANES 2007-2012. *BMJ Open Diabetes Res. Care* **4**, e000154 (2016).
23. SEER. Cancer of the Colon and Rectum - Cancer Stat Facts. *SEER* https://seer.cancer.gov/statfacts/html/colorect.html (2023).
24. Liu, Y., Carlson, S. A., Watson, K. B., Xu, F. & Greenlund, K. J. Trends in the prevalence of chronic obstructive pulmonary disease among adults aged ≥18 years - United States, 2011-2021. *MMWR Morb. Mortal. Wkly. Rep.* **72**, 1250–1256 (2023).
25. Jin, J.-M. *et al.* Gender differences in patients with COVID-19: Focus on severity and mortality. *Front. Public Health* **8**, 152 (2020).
26. Muzic, J. G. *et al.* Incidence and trends of basal cell carcinoma and cutaneous squamous cell carcinoma: A population-based study in Olmsted county, Minnesota, 2000 to 2010. *Mayo Clin. Proc.* **92**, 890–898 (2017).
27. White, R. H. The epidemiology of venous thromboembolism. *Circulation* **107**, I4-8 (2003).
28. Gwira, J. A., Fryar, C. D. & Gu, Q. Prevalence of total, diagnosed, and undiagnosed diabetes in adults: United States, August 2021-August 2023. *NCHS Data Brief* (2024) doi:10.15620/cdc/165794.
29. Bullard, K. M. *et al.* Prevalence of diagnosed diabetes in adults by diabetes type - United States, 2016. *MMWR Morb. Mortal. Wkly. Rep.* **67**, 359–361 (2018).
30. SEER. Diffuse Large B-Cell Lymphoma - Cancer Stat Facts. *SEER* https://seer.cancer.gov/statfacts/html/dlbcl.html (2023).
31. Varma, R. *et al.* Prevalence of and risk factors for diabetic macular edema in the United States. *JAMA Ophthalmol.* **132**, 1334–1340 (2014).
32. Rein, D. B. *et al.* Prevalence of age-related macular degeneration in the US in 2019. *JAMA Ophthalmol.* **140**, 1202–1208 (2022).
33. Soo, R. A. *et al.* Prevalence of EGFR mutations in patients with resected stages I to III NSCLC: Results from the EARLY-EGFR study. *J. Thorac. Oncol.* **19**, 1449–1459 (2024).
34. Zhu, Y., Pandya, B. J. & Choi, H. K. Prevalence of gout and hyperuricemia in the US general population: the National Health and Nutrition Examination Survey 2007-2008. *Arthritis Rheum.* **63**, 3136–3141 (2011).

35. Grimberg, A. *et al.* Gender bias in U.s. pediatric growth hormone treatment. *Sci. Rep.* **5**, 11099 (2015).

36. Bradley, H. *et al.* Hepatitis C virus prevalence in 50 U.s. states and D.c. by sex, birth cohort, and race: 2013-2016. *Hepatol. Commun.* **4**, 355–370 (2020).

37. Mulder, J. W. C. M. *et al.* Sex differences in diagnosis, treatment, and cardiovascular outcomes in homozygous familial hypercholesterolemia. *JAMA Cardiol.* **9**, 313–322 (2024).

38. Ibrahim, A., Morais, S., Ferro, A., Lunet, N. & Peleteiro, B. Sex-differences in the prevalence of Helicobacter pylori infection in pediatric and adult populations: Systematic review and meta-analysis of 244 studies. *Dig. Liver Dis.* **49**, 742–749 (2017).

39. Gov, H. I. V. U.S. Statistics. *Hiv.gov* https://www.hiv.gov/hiv-basics/overview/data-and-trends/statistics (2025).

40. de Miguel-Yanes, J. M. *et al.* Incidence, outcomes and sex-related disparities in pneumonia: A matched-pair analysis with data from Spanish hospitals (2016-2019). *J. Clin. Med.* **10**, 4339 (2021).

41. Humphrey, T. *et al.* How common is hyperkalaemia? A systematic review and meta-analysis of the prevalence and incidence of hyperkalaemia reported in observational studies. *Clin. Kidney J.* **15**, 727–737 (2022).

42. Rosen, R. C. Prevalence and risk factors of sexual dysfunction in men and women. *Curr. Psychiatry Rep.* **2**, 189–195 (2000).

43. Kautzky-Willer, A., Kosi, L., Lin, J. & Mihaljevic, R. Gender-based differences in glycaemic control and hypoglycaemia prevalence in patients with type 2 diabetes: results from patient-level pooled data of six randomized controlled trials. *Diabetes Obes. Metab.* **17**, 533–540 (2015).

44. Kim, Y. S. & Kim, N. Sex-gender differences in irritable bowel syndrome. *J. Neurogastroenterol. Motil.* **24**, 544–558 (2018).

45. Zheng, Y. *et al.* Fatal infections among cancer patients: A population-based study in the United States. *Infect. Dis. Ther.* **10**, 871–895 (2021).

46. Adjaye-Gbewonyo, D., Ng, A. & Black, L. *Sleep Difficulties in Adults: United States, 2020*. http://dx.doi.org/10.15620/cdc:117490 (2022) doi:10.15620/cdc:117490.

47. Williams, A., Ansai, N., Ahluwalia, N. & Nguyen, D. *Anemia Prevalence: United States, August 2021- August 2023*. http://dx.doi.org/10.15620/cdc/168890 (2024) doi:10.15620/cdc/168890.

48. Elflein, J. Prevalence of inflammatory bowel disease in the U.S. in 2015-2016, by gender. *Statista* https://www.statista.com/statistics/772769/inflammatory-bowel-disease-prevalence-us-by-gender/.

49. Nguyen, D. Q. *et al.* Temporal trends and racial/ethnic- and sex-differences in LDL cholesterol control among US adults with self-reported atherosclerotic cardiovascular disease. *Am. J. Prev. Cardiol.* **18**, 100673 (2024).

50. Hocaoğlu, M. *et al.* Incidence, prevalence, and mortality of lupus nephritis: A population-based study over four decades using the lupus Midwest network. *Arthritis Rheumatol.* **75**, 567–573 (2023).

51. NIMH. Major Depression. *National Institute of Mental Health (NIMH)* https://www.nimh.nih.gov/health/statistics/major-depression (2021).

52. Burch, R., Rizzoli, P. & Loder, E. The prevalence and impact of migraine and severe headache in the United States: Figures and trends from government health studies. *Headache* **58**, 496–505 (2018).

53. Dilokthornsakul, P. *et al.* Multiple sclerosis prevalence in the United States commercially insured population. *Neurology* **86**, 1014–1021 (2016).

54. Zucker, D. R., Griffith, J. L., Beshansky, J. R. & Selker, H. P. Presentations of acute myocardial infarction in men and women. *J. Gen. Intern. Med.* **12**, 79–87 (1997).

55. Fabling, J. M. *et al.* Postoperative nausea and vomiting. A retrospective analysis in patients undergoing elective craniotomy. *J. Neurosurg. Anesthesiol.* **9**, 308–312 (1997).

56. Hilarius, D. L. *et al.* Chemotherapy-induced nausea and vomiting in daily clinical practice: a community hospital-based study. *Support. Care Cancer* **20**, 107–117 (2012).

57. Lambert, L. A. *et al.* Incidence, risk factors, and impact of severe neutropenia after hyperthermic intraperitoneal mitomycin C. *Ann. Surg. Oncol.* **16**, 2181–2187 (2009).

58. Kapetanakis, V. V. *et al.* Global variations and time trends in the prevalence of primary open angle glaucoma (POAG): a systematic review and meta-analysis. *Br. J. Ophthalmol.* **100**, 86–93 (2016).

59. Ducrotté, P., Milce, J., Soufflet, C. & Fabry, C. Prevalence and clinical features of opioid-induced constipation in the general population: A French study of 15,000 individuals. *United European Gastroenterol. J.* **5**, 588–600 (2017).

60. Sarafrazi, N. *Osteoporosis or Low Bone Mass in Older Adults: United States, 2017-2018*. http://dx.doi.org/10.15620/cdc:103477 (2021) doi:10.15620/cdc:103477.

61. Stewart, W. F. *et al.* Prevalence and burden of overactive bladder in the United States. *World J. Urol.* **20**, 327–336 (2003).

62. Carla E. Zelaya, Ph.D., James M. Dahlhamer, Ph.D., Jacqueline W. Lucas, M.P.H., and Eric M. Connor, B.S. Chronic Pain and High-impact Chronic Pain Among U.S. Adults, 2019 NCHS Data Brief 2020. https://www.cdc.gov/nchs/products/databriefs/db390.htm (2020).

63. CDCMMWR. QuickStats: Age-adjusted death rates* for Parkinson disease† among adults aged ≥65 years - national vital statistics system, United States, 1999-2017. *MMWR Morb. Mortal. Wkly. Rep.* **68**, 773 (2019).

64. Kobau, R., Luncheon, C. & Greenlund, K. Active epilepsy prevalence among U.S. adults is 1.1% and differs by educational level-National Health Interview Survey, United States, 2021. *Epilepsy Behav.* **142**, 109180 (2023).

65. Armstrong, A. W. *et al.* Psoriasis prevalence in adults in the United States. *JAMA Dermatol.* **157**, 940–946 (2021).

66. Osakwe, N. & Hashmi, M. F. Uremic pruritus evaluation and treatment. in *StatPearls* (StatPearls Publishing, Treasure Island (FL), 2025).

67. Stang, C. D. *et al.* Incidence, prevalence, and mortality of psychosis associated with Parkinson's disease (1991-2010). *J. Parkinsons. Dis.* **12**, 1319–1327 (2022).

68. SEER. Cancer of the Kidney and Renal Pelvis - Cancer Stat Facts. *SEER* https://seer.cancer.gov/statfacts/html/kidrp.html (2023).

69. Adamus, M., Gabrhelik, T. & Marek, O. Influence of gender on the course of neuromuscular block following a single bolus dose of cisatracurium or rocuronium. *Eur. J. Anaesthesiol.* **25**, 589–595 (2008).

70. CDC. Rheumatoid Arthritis. *https://www.cdc.gov/arthritis/rheumatoid-arthritis/index.html* https://www.cdc.gov/arthritis/rheumatoid-arthritis/index.html (2025).

71. Arnson, Y. *et al.* Comparing management and outcomes in men and women with nonvalvular atrial fibrillation: Data from a population-based cohort. *JACC Clin. Electrophysiol.* **4**, 604–614 (2018).

72. Havers, F. P. *et al.* Characteristics and outcomes among adults aged ≥60 years hospitalized with laboratory-confirmed Respiratory Syncytial Virus - RSV-NET, 12 states, July 2022-June 2023. *MMWR Morb. Mortal. Wkly. Rep.* **72**, 1075–1082 (2023).

73. Li, R., Ma, X., Wang, G., Yang, J. & Wang, C. Why sex differences in schizophrenia? *J. Transl. Neurosci. (Beijing)* **1**, 37–42 (2016).

74. CDC. Chronic Kidney Disease in the United States, 2023. *Chronic Kidney Disease* https://www.cdc.gov/kidney-disease/php/data-research/index.html (2025).

75. Izmirly, P. M. *et al.* Prevalence of systemic lupus erythematosus in the United States: Estimates from a meta-analysis of the Centers for Disease Control and Prevention national lupus registries. *Arthritis Rheumatol.* **73**, 991–996 (2021).

76. Yassa, R. & Jeste, D. V. Gender differences in tardive dyskinesia: a critical review of the literature. *Schizophr. Bull.* **18**, 701–715 (1992).

77. Schoonen, W. M. *et al.* Epidemiology of immune thrombocytopenic purpura in the General Practice Research Database. *Br. J. Haematol.* **145**, 235–244 (2009).

78. Belderok, S.-M., van den Hoek, A., Kint, J. A., Schim van der Loeff, M. F. & Sonder, G. J. Incidence, risk factors and treatment of diarrhoea among Dutch travellers: reasons not to routinely prescribe antibiotics. *BMC Infect. Dis.* **11**, 295 (2011).

79. Lodise, T. P., Manjelievskaia, J., Marchlewicz, E. H. & Rodriguez, M. Retrospective cohort study of the 12-month epidemiology, treatment patterns, outcomes, and health care costs among adult patients with complicated urinary tract infections. *Open Forum Infect. Dis.* **9**, ofac307 (2022).
