# Supplementary material for: Sex representation in trials relative to indication-specific disease burden in FDA-approved drugs (2015–2023)
Source: Nat Commun. 2026 Jun 23;17:6962. doi: 10.1038/s41467-026-74469-z (PMC13392115; doi:10.1038/s41467-026-74469-z)
Supplement: Supplementary file 9 — Transparent Peer Review file [file 41467_2026_74469_MOESM9_ESM.pdf]

nature portfolio

Peer Review File

Sex Representation in Trials Relative to Indication-Specific Disease Burden in FDA-Approved Drugs (2015–2023)

Corresponding Author: Dr Sophie Zaaijer

This file contains all reviewer reports in order by version, followed by all author rebuttals in order by version.

Version 0:

Reviewer comments:

Reviewer #1

(Remarks to the Author)
Thank you for the opportunity to review this manuscript. The questions asked in this study respond to a significant amount of distortion and misrepresentation in the debate over whether current resource allocations in biomedical science are appropriate given a long history of androcentrism in medical research. This study argues that in the US (1) over the last decade, female predominant conditions have outnumbered male predominant conditions in drug approvals, and (2) there has been an increase in the number of trials in which enrollment numbers align with the gender/sex distribution of disease prevalence in the general population, though there remain some notable areas where men and women are underrepresented relative to the distribution of impact.

The weakness of this manuscript lies in (1) the lack of proper setting of these findings within the literature streams and scientific debates over recent decades about the heterogeneous advance of the inclusion of women's specific conditions and needs in medical science, and relatedly (2) the lack of critical reflection and conceptual discussion about what would constitute the right measures and benchmarks for "progress" in commitment to women's health in biomedicine.

These problems are crystallized by the problematic core assumptions of the study, for example, that gender equity should be represented by clinical trial representation that is equal to the "prevalence" of the condition in the population, and that a condition that has a prevalence of 75% or greater for one gender/sex represents a "predominant" condition for that sex. On the first matter, it should be incidence, not prevalence, that guides our consideration given considerable age-related demographic differences in the gendered structure of the population. For example, ADRDs are more prevalent in women, but the incidence is the same for men and women - and this is because there are considerably more older women.

The 75% prevalence cut-off is further problematic because of the asymmetry in the number of conditions that would ever meet this threshold for men, as illustrated by the authors' data table/Figure 1. There are many reasons for this - including the flawed use of prevalence vs incidence, but also including the very significant gendered social patterns in which women in the US are much higher users of medical systems and tend to receive multiple diagnoses along a pathway of care (eg, Lee et al. 2023 "A Gender Hypothesis"). An indication that the authors have not immersed themselves in the relevant literature on gender, health, and medicine is that they do not consider the likelihood that female predominant indication drug approvals reflect a more cynical trend to define new female-specific diagnoses in order to exploit the female-specific market.

Equally problematic is the out of date framing of the study benchmarks as a simple binary comparison between the representation of women and men in medical science. In studies of gender and health in the US context it is vital and cardinal to include subgroup analysis within these categories (eg, Krieger 2018, "US black and white women born 1915-1979") because the "equal" representation of men and women can obscure significant distortions in the representation of particular subgroups of men and of women that would cause us to revise our understanding of gendered disparities in inclusion and representation in medical science.

Thus, while I think the empirical work undertaken in this study has potential to add to the literature, it is not publication-ready and needs significant rethinking and contextualization in the appropriate literature to ensure that appropriate inferences are made from the authors' findings.

Reviewer #2

(Remarks to the Author)
Thank you for giving me the opportunity to review this interesting manuscript by Zaaijer and Groen. The authors analyzed 187 FDA approved drugs to investigate potential sex/gender biases in allocation of approval and in representation of female participants in the studies. According to their classification they identify an increase in trials for female-predominant conditions but a persistence of enrollment disparities.

Overall, this is an important and timely analysis that addresses a persistent health disparity. The numbers are interesting, but I need to note a number of potential sources of skewed representation given the classifications employed. I would urge the authors to at least clarify many of these point in the text, if not correct for them in the analysis.

1. While it appears laudable that more attention is being devoted to female-predominant conditions, this group is quite heterogeneous in the current analysis. The authors group gynecological conditions (which by definition will rarely include gender-diverse individuals in their trials and do, hence, often exclusively include biologically female participants) and lump them together with non-gynecological conditions, where a selective choice to include female participants in adequate numbers has to be made. For the sake of clarity, it would be important to differentiate between e.g. treatment for vulvovaginal candidiasis, endometrial and prostate cancer, or endometriosis and diseases that affect both female and male patients such as multiple sclerosis. The mechanisms of inclusion for these conditions are very different and rely on different approaches by the investigator, and hence, these should be reported separately.
2. The indicated prevalence rates of the conditions appear very US-skewed. This is especially obvious in the case of HIV, which is declared a significantly male-dominated diseases. This might be true in the US, but does definitely not apply in the same manner to the prevalence of HIV at a global level. Since FDA approval is relevant for the US, but many of these conditions have global impacts, global prevalence should probably be used to define their sex-specific distributions.
3. The reported from line 65 on should account for some of the skewed distributions. E.g. of the 13 studies reported for female-predominant cancers, 11 alone are for breast cancer. The current depiction does not clearly point this out, although it is an important aspect to consider when placing the numbers into a larger context. Overrepresentation of some indications in the list should be clearly stated, as this gives insight into funding, testing and approval patterns.
4. Last, I like the graphical representation, however, I am left to wonder again, if the number should not be corrected for trials that by definition do only include female or male participants. These could be shown in the figure, but could possibly be removed from the overall calculation, as they are an outlier compared to the general distribution. An alternative would be to compare the numbers with their inclusion and without, giving a clear visual of the effect of these one-sex studies on the general analysis.

Version 1:

Reviewer comments:

Reviewer #2

(Remarks to the Author)
Thanks for giving me the opportunity to re-review this manuscript by Zaaijer and Groen. I feel this extended version allows for more context and nuance and significantly improves the manuscript correcting some of the simplifications present in the short version. The authors have done a good job in placing the results in a broader – and dynamic – context and have expanded on the figures providing a more detailed and precise representation of the results.
Furthermore, I think the reviewer comments have been addressed and productively incorporated.

I only have two minor points:
From line 180 on, in the discussion of breast vs prostate cancer, the element of mean age at diagnosis and diagnostic spread is not taken into consideration. Breast cancer is diagnosed on average at 62 years and prostate cancer at 67 years, in addition to this difference the spread of these diagnoses is somewhat wider for breast cancer with a significant number of younger patients being diagnosed who profit immensely from early detection and therapy. The current phrasing could be interpreted as if the mayor investment in breast cancer research was somewhat unjustified compared to prostate cancer research. Maybe this point could be added and the whole paragraph potentially slightly adapted.

For figure 2 – what is “cancer associated”?

Reviewer #3

(Remarks to the Author)
Thank you for the opportunity to review the manuscript by Zaaijer and Groen. The research addresses an important and timely question; however, I have substantial concerns regarding the methodological approach to data analysis and interpretation.

1. Data characterization
I find the analyses presented in the "Data Characterization" section and Figure 2 (with supporting Table S2-1) to be methodologically problematic. The authors should define the studied conditions that permits meaningful clinical

interpretation of sex-based differences. For example, gynecological conditions should be excluded, as the study population consists exclusively of women by design; their inclusion alongside sex-agnostic conditions such as hyposexuality conflates distinct phenomena and obscures interpretability. Similarly, sex-predominant malignancies—including prostate, breast, ovarian, and cervical cancers—should be stratified separately or excluded to enable readers to draw valid inferences about disease representation. I recommend applying this stratification principle systematically across all reported analyses.

2. Statistical testing and multiple comparisons
The use of chi-squared tests at a nominal significance threshold ($p < 0.05$) to assess sex representation within clinical trials against disease prevalence is potentially problematic given the number of hypothesis tests conducted in this study. The authors should address multiple hypothesis testing through rigorous correction methods such as Bonferroni adjustment, the Benjamini-Hochberg procedure, or the Holm method, rather than proceeding without correction. This is important for controlling the family-wise error rate and ensuring the reliability of reported findings.

3. The use of LLM for data standardization
I have concerns about the reliance on Perplexity AI's natural language processing for data standardization. While LLM-based preprocessing may serve as a valuable initial step, the approach requires comprehensive human (manual) validation to ensure accuracy and fidelity. Large language models may exhibit variable performance across different clinical conditions—performing reliably for certain diagnoses while potentially underperforming for others—which risks introducing systematic bias into the dataset and downstream conclusions.

The revision shows that the authors have substantially strengthened several aspects of the manuscript. The expanded Discussion, which now addresses issues such as portfolio focus, enrollment proportionality, reproductive risks, endpoint and diagnostic complexity, and sponsorship and oversight, goes a long way toward remedying the original lack of contextualization within the literature on women's health and clinical trial representation. The change from an arbitrary 75% prevalence cutoff to a standard deviation–based classification around the US Census mean is also a clear methodological improvement that better reflects variation in sex-specific disease burden across indications.

However, some of the core conceptual concerns raised by the reviewer remain only partially resolved. A central issue is the question the reviewer posed about what should properly count as progress in commitment to women's health, and what benchmarks are appropriate to assess it. The authors now justify the use of prevalence and incidence more carefully and give a cogent rationale for using prevalence as the denominator for chronic conditions such as ADRD, where prevalence better captures the pool of patients eligible for trials. Yet the manuscript still tends to conflate alignment of enrollment with sex-disaggregated disease burden, within a binary sex framework, with equity or commitment. Proportional enrollment is a useful metric, but it is only one possible benchmark and it is sensitive to the diagnostic and healthcare utilization processes that generate prevalence estimates in the first place. The authors do acknowledge that women's greater healthcare utilization can inflate prevalence and that male-phenotype-based diagnostic criteria can under-recognize disease in women, but they stop short of drawing out the full implication: that their chosen benchmark is itself shaped by gendered social and clinical practices, and that "alignment with prevalence" cannot be treated as a straightforward measure of fairness or equity. A more explicit reflection on why this particular benchmark was chosen, what its limitations are as a measure of progress, and how it might diverge from other plausible benchmarks would strengthen the conceptual core of the paper.

On incidence versus prevalence, the authors' revision is a clear improvement. They now distinguish thoughtfully between incidence and prevalence, invoke CDC definitions, and treat chronic versus acute conditions differently. The justification for using prevalence for ADRD, in my view, is reasonable. Nonetheless, because the reviewer's concern was not merely technical but tied to broader demographic and age-structure issues, it would help if the manuscript more openly acknowledged that different choices of denominator can yield different pictures of burden and representation and that what counts as appropriate depends on the normative question one is asking. The methodological decision to use prevalence for certain conditions should be explicitly connected back to the conceptual question of what commitment and progress mean in this context.

The question of intersectionality is only partially addressed. The authors point out that the FDA DTSP data structure allows only binary sex and that sex-by-race interactions are not reported, and it cites the Bierer paper to show that this is a recognized limitation. However, the manuscript itself does not adequately convey how serious a conceptual limitation this is for any claims about equity. The analyses aggregate all women into a single category and then interpret improvements in aggregate female representation as evidence of progress. The reviewer's point was that such aggregation can "obscure significant distortions" for particular subgroups of women and men. The manuscript would benefit from explicitly stating that its binary sex analysis cannot show whether improvements apply across racial and socioeconomic subgroups, and that claims about "equity" and "commitment to women's health" should therefore be read as applying only at the most aggregated level. This limitation should be clearly flagged in the main Discussion where the authors interpret their findings, not just in the Methods or Limitations, and it should be explicitly connected to the reviewer's concern about subgroup disparities.

The framing of the paper, especially in the title and some strong statements in the Discussion, still presents the results in terms that are more definitive than the data justify. Phrases that the study "demonstrates the level of industry's commitment to women's health" and that industry is "meaningfully investing in lowering female disease burden" go beyond what can be established from patterns of approvals and trial enrollment alone. The reviewer's original concern about "the right measures and benchmarks for 'progress' in commitment to women's health" has not yet been fully absorbed into the framing. It would be more accurate to describe the study as assessing patterns in approvals and enrollment that are consistent with increased attention to conditions with a higher female disease burden and with more proportional inclusion of women in some areas, while also documenting areas where women remain underrepresented. The interpretive language should be recalibrated

from strong claims about commitment and progress to more modest claims about what the observed distributions can and cannot tell us.

Finally, the manuscript still tends to slide between “sex” and “gender” language, despite the authors’ acknowledgment that the FDA captures binary sex and not gender identity. This was part of the reviewer’s broader critique that the manuscript’s framing as a binary comparison between “men” and “women” is out of date in contemporary gender and health research. To respond fully, the authors should not only clarify terminologically that they are analyzing sex as recorded in regulatory documents, but also reflect briefly on what is missing from this picture: gender-diverse individuals, the social dimensions of gender, and intersectional positions that cannot be captured by a binary variable. This reflection should be connected back to the reviewer’s point that progress in representation cannot be adequately assessed with a strictly binary framework and that the study’s conclusions about “gender equity” should therefore be carefully qualified.

Version 2:

Reviewer comments:

Reviewer #2

(Remarks to the Author)
All comments have been addressed.

Reviewer #3

(Remarks to the Author)
I thank the authors for their detailed responses and for the substantial effort invested in this revision. The implementation of Benjamini-Hochberg correction, the shift to a SD-based prevalence classification, the stratification of sex-specific from sex-predominant indications, the clarification of the LLM validation workflow, and the expanded discussion all represent improvements. Despite these improvements, several core conceptual concerns remain only partially resolved.

First, I previously pointed out that "alignment with prevalence" cannot be treated as a straightforward measure of fairness, because the prevalence benchmarks themselves are shaped by gendered diagnostic practices and differential healthcare utilization. The authors have added an acknowledgment of this in the limitations section (lines 437–444), which I appreciate. However, the discussion continues to frame the 67% alignment finding as "an encouraging signal that suggests conscious efforts toward female inclusion" without any qualifier/justification. Most readers will engage primarily with the discussion, not the limitations, and will encounter the central finding decontextualized/misunderstood. I ask the authors to add two to three sentences directly to the discussion, adjacent to the 67% interpretation, acknowledging that "alignment with prevalence" should be read as a pragmatic rather than normative measure of representation.

Second, I requested that the intersectionality limitation be flagged in the main discussion where findings are interpreted, not only in limitations. The paragraph added at lines 464–471 is welcome but does not fulfill this request. Positive findings for oncology and neurology enrollment are interpreted in the discussion without any qualifier that these gains apply only at the most aggregated level of binary biological sex and may not reflect equitable participation across racial, ethnic, and socioeconomic subgroups of women. I ask for a brief qualifier, at minimum at the close of the "Enrollment Proportionality" subsection, making this clearer. The Limitations paragraph should remain, but cannot substitute for an in-Discussion acknowledgment.

Third, the title change from "commitment" to "attention" is welcome. However, the phrase "an encouraging signal that suggests conscious efforts toward female inclusion" (lines 202–205) attributes deliberate industry agency to observational distributional patterns, which the study design cannot establish. I ask the authors to replace intent-implying phrases with pattern-based language throughout. For example, "patterns consistent with increased female inclusion" in place of "conscious efforts toward female inclusion." This is a request for language precision appropriate to an observational study, not a request to weaken its message.

Fourth, the discussion presents proportional enrollment as an achievement without drawing out a further implication: if the benchmark is inflated by women's higher healthcare utilization, alignment with it may represent alignment with a floor rather than a meaningful ceiling of appropriate inclusion. Conversely, where diagnostic criteria are male-calibrated, as in cardiovascular disease, "alignment" may reflect alignment with an underestimate of the true female disease burden. I ask for one to two sentences in the Discussion making this circular dependency explicit.

Finally, despite acknowledging that the FDA DTSP captures binary biological sex rather than gender identity, the manuscript continues to intermittently use "gender" where "sex" is the correct term. A thorough, line-by-line audit is required before this manuscript can be considered terminologically consistent with its own epistemological framing.

Minor points:
1. The temporal regression ($F_{1,7} = 0.0142$, $P = 0.909$) is presented as showing "no change across years," but nine annual observations provide insufficient power to support this conclusion. The finding should be reframed as "no statistically detectable trend," with an explicit acknowledgment that absence of evidence is not evidence of absence.

2. The two osteoporosis trials enrolling 100% women are noted but not examined. For internal consistency with the paper's

equity framing, the reciprocal exclusion of men, who account for approximately 18% of osteoporosis cases, warrants at least brief comment.

3. Finally, the survivorship bias hypothesis (lines 406–425) is a material confounder for the 67% alignment claim and should be noted in the Abstract so that readers do not encounter the central finding without this important qualification.

Version 3:

Reviewer comments:

Reviewer #3

(Remarks to the Author)
I thank you for your continued engagement with my concerns and for the considerable effort invested in this second revision. The manuscript has improved meaningfully across several dimensions. The implementation of Benjamini-Hochberg correction is appropriate and clearly documented throughout Table S4. The shift to SD-based prevalence thresholds provides a principled and reproducible classification framework. The stratification of sex-specific from sex-predominant indications is now cleanly and consistently applied, which is a critical methodological improvement. The expanded Discussion on cardiovascular and autoimmune under-enrollment is substantive and adds genuine interpretive value. Despite these improvements, several concerns remain insufficiently resolved, and I cannot recommend acceptance in the current form.

My first concern relates to the use of prevalence alignment as a fairness metric. In my previous review, I raised the concern that prevalence benchmarks are themselves shaped by gendered diagnostic practices and differential healthcare utilization, and therefore cannot be treated as a proxy for equitable representation. Authors have added a brief acknowledgment in the limitations section (lines 437–444), which I appreciate. However, the Discussion continues to frame the 67% alignment figure in broadly positive terms without adequately contextualizing this caveat. Alignment with prevalence is a necessary but not sufficient condition for equity. In conditions historically underdiagnosed in women, including cardiovascular disease, ADHD, and certain autoimmune disorders, the prevalence benchmark itself likely undercounts the true female disease burden. Framing alignment with such a benchmark as a marker of progress risks perpetuating the very bias the study aims to expose. I ask that you more explicitly acknowledge this in the Discussion, not merely in the limitations section, and temper the positive framing of the 67% figure accordingly.

My second concern relates to the temporal trend analysis. The manuscript reports that no change in alignment was observed across the years and presents this as a substantive finding. However, with approximately 195 drugs approved over 9 years (roughly 22 per year), the annual sample sizes are small. As the analysis is probably under-powered, a null finding in a low-powered analysis cannot be interpreted as evidence of a true absence of temporal change. This limitation is not acknowledged anywhere in the manuscript. I ask that you either explicitly state that the temporal null finding may reflect insufficient statistical power rather than a true absence of trend, or provide a post-hoc power calculation or sensitivity analysis to support the claim.

My third concern relates to the LLM validation workflow. The clarification provided is an improvement over the prior version. However, the Methods section still lacks the specificity needed for independent reproducibility. In particular, it is not reported which specific data extraction or classification tasks the LLM was applied to, how LLM outputs were validated against a human-curated ground truth, and what discordance or error rate was observed. Given that LLM-assisted data processing is not yet a standardized practice in biomedical research, this level of transparency is essential. I ask that you add a concise but complete description of these three elements.

My fourth concern relates to the title. The current phrasing, "...Demonstrates the Level of Industry's Attention to Women's Health," remains ambiguous. While I understand you may intend this as a neutral descriptor, it risks being read as broadly congratulatory given the persistent gaps documented in the manuscript. A more precise formulation that reflects both the progress and the structural barriers identified would better represent the study's nuanced conclusions and avoid mischaracterization in citation contexts. I encourage revision.

Open Access This Peer Review File is licensed under a Creative Commons Attribution 4.0 International License, which permits use, sharing, adaptation, distribution and reproduction in any medium or format, as long as you give appropriate credit to the original author(s) and the source, provide a link to the Creative Commons license, and indicate if changes were made.
In cases where reviewers are anonymous, credit should be given to 'Anonymous Referee' and the source.
The images or other third party material in this Peer Review File are included in the article's Creative Commons license, unless indicated otherwise in a credit line to the material. If material is not included in the article's Creative Commons license and your intended use is not permitted by statutory regulation or exceeds the permitted use, you will need to obtain permission directly from the copyright holder.
To view a copy of this license, visit https://creativecommons.org/licenses/by/4.0/

Point-by-point response to the reviewers' comments:

Analysis of 195 US FDA-Approved Drugs (2015–2023) across 98 Unique Indications Demonstrates the Level of Industry's Commitment to Women's Health

Sophie Zaaijer[1,2,3*], Simon C. Groen[1*]

We thank the Editor and Reviewers for their thoughtful and constructive comments. We have now addressed these by implementing extensive revisions in our manuscript.

Our manuscript was originally submitted to a related *Nature* family journal with a different article structure. We are grateful for the opportunity to adapt it to *Nature Communications*' format, which allowed us to enrich the manuscript with clearer context, present the results of our analyses in a tighter narrative, and extend the Discussion section to place our interpretations of the results in the broader landscape of existing literature in the field.

We have addressed every comment in depth and provide extended analyses, clarifications, and citations—we viewed this revision as a welcome and valuable opportunity to strengthen the presentation of our work.

Below we provide a point-by-point response to each comment and describe the corresponding revisions. Unless otherwise noted, all page and line numbers refer to the revised manuscript.

Reviewer #1 (Remarks to the Author):

Thank you for the opportunity to review this manuscript. The questions asked in this study respond to a significant amount of distortion and misrepresentation in the debate over whether current resource allocations in biomedical science are appropriate given a long history of androcentrism in medical research.

This study argues that in the US (1) over the last decade, female predominant conditions have outnumbered male predominant conditions in drug approvals, and (2) there has been an increase in the number of trials in which enrollment numbers align with the gender/sex distribution of disease prevalence in the general population, though there remain some notable areas where men and women are underrepresented relative to the distribution of impact.

The weakness of this manuscript lies in (1) the lack of proper setting of these findings within the literature streams and scientific debates over recent decades about the heterogeneous advance of the inclusion of women's specific conditions and needs in medical science, and relatedly (2) the lack of critical reflection and conceptual discussion about what would constitute the right measures and benchmarks for "progress" in commitment to women's health in biomedicine.

We thank the reviewer for their engagement with our manuscript. We recognize and share the commitment to women's health in biomedicine.

We agree with the reviewer that our initial submission was lacking in properly placing the results of our analyses in context of the literature. This can be explained by its history: it was originally intended as a short-form article for a parallel *Nature* family journal but was eventually transferred to *Nature Communications*.

Our updated manuscript now contains a more extensive Discussion section in which we place our findings within the literature streams and scientific debates over recent decades and critically reflect on what would constitute the right measures and benchmarks for "progress" in this area of health.

The outline of topics in our Discussion section is now as follows:

- Focus of drug portfolios
- Enrollment proportionality
- Oncology as exemplar
- Mitigation of reproductive risks
- Endpoint complexity
- Diagnostic complexity
- Design of drug development pipelines
- Sponsorship & oversight
- “Inclusion & success” hypothesis (survivorship; variance/false negatives; regulatory delays)
- Limitations (prevalence vs. incidence; geography; sub-indication heterogeneity)

These problems are crystallized by the problematic core assumptions of the study, for example, that gender equity should be represented by clinical trial representation that is equal to the "prevalence" of the condition in the population, and that a condition that has a prevalence of 75% or greater for one gender/sex represents a "predominant" condition for that sex.

On the first matter, it should be incidence, not prevalence, that guides our consideration given considerable age-related demographic differences in the gendered structure of the population. For example, ADRDs are more prevalent in women, but the incidence is the same for men and women - and this is because there are considerably more older women.

Like the reviewer, we recognize that the incidence and prevalence of a condition are two different metrics of disease burden across a population. In our study we followed the definitions of incidence and prevalence as explained by the Centers of Disease Control and Prevention in their Principles of Epidemiology.

We have now incorporated these definitions in the Methods and address limitations of having to use both incidence and prevalence estimates in a paragraph entitled "Limitations" in the Discussion:

Methods: LINES 521-530
For each indication we chose the epidemiologically appropriate denominator—prevalence for chronic/relapsing conditions, incidence for acute diseases—selecting the most conservative estimate when sources conflicted and assessing study quality (sample size, case definition, bias, timeframe, representativeness).
[…]
All values were harmonized to %W|D (percent women among affected); we use "prevalence" as shorthand and note when incidence is used."

Discussion – LINES 423-428
"*First, our estimates of sex-specific disease burden draw on both prevalence and incidence data, which capture different aspects of disease biology and healthcare access. Prevalence is the current burden; incidence counts new cases over time. Chronic conditions are typically measured by prevalence, acute events by incidence[64]. Prevalence and incidence measures are therefore not directly comparable, although our use of sex ratios mitigates some potential biases."*

A specific note on ADRDs:

We appreciate the reviewer's use of age-related conditions such as Alzheimer's disease and related dementias (ADRD) in this context. For these conditions, prevalence can conflate risk of onset with chance of survival, as women on average live longer than men. Recent syntheses (e.g., Huque et al. [*J Alzheimers Dis.*, 2023] and a report from the Alzheimer's Association) show that while ADRD ***incidence*** is similar by sex, ADRD ***prevalence*** is higher among women, consistent with a longer typical duration of disease in a woman.

For our study's aim—benchmarking trial enrollment against the population that stands to benefit from a new therapy—prevalence is the more policy-relevant denominator: it reflects the number of people living with ADRD who could be recruited into clinical trials and assessed for treatment safety and efficacy over time. We therefore use prevalence disaggregated by sex as our metric for ADRD burden across the population.

We have now addressed this in the revised manuscript by inserting the following sentence to the Methods: LINE 524-527: "*For example Alzheimer's disease we benchmarked enrollment to sex-disaggregated prevalence (not incidence). Because women live longer, their share of prevalence exceeds incidence. Prevalence better reflects those living with disease—i.e., eligible for trials and longest exposure to potential benefits and risks of a new drug."*

Furthermore, we added a section entitled "Limitations" to the Discussion, which outlines several limitations of having to combine epidemiological data from different sources.

The 75% prevalence cut-off is further problematic because of the asymmetry in the number of conditions that would ever meet this threshold for men, as illustrated by the authors' data table/Figure 1.

We agree with the reviewer that using a single 75% prevalence cut-off may be problematic. In light of the reviewer's comment, we have now updated the analysis by binning disease indications based on how far the percentage of females in the patient population are removed from the percentages of females in the US census population. Bins are sized by incremental numbers of standard deviations above or below the US census number with indications below being classed as male-biased disease indications and indications above being classed as female-biased disease indications. We describe the revised analysis in the following sections of the manuscript:

Results – LINE 111-116*: "Over the nine-year period evaluated, most approvals involved therapies for indications that affect both sexes, with 56% (n=121) of drugs treating indications with a sex-based prevalence that fell within ±1 SD of the US Census mean (50.5%). Indications with markedly higher female prevalence (≥2 SD [≥67.167% and <83.8%] and ≥3 SD [≥83.8%] removed from the US Census mean) had treatments approved by the FDA twice as frequent (n=65) as those that predominantly affect males in the corresponding disease prevalence ranges (n=32;* ***Fig. 3, Table S2-2****)."*

There are many reasons for this - including the flawed use of prevalence vs incidence, but also including the very significant gendered social patterns in which women in the US are much higher users of medical systems and tend to receive multiple diagnoses along a pathway of care (eg, Lee et al. 2023 "A Gender Hypothesis"). An indication that the authors have not immersed themselves in the relevant literature on gender, health, and medicine is that **they do not consider the likelihood that female predominant indication drug approvals reflect a more cynical trend to define new female-specific diagnoses in order to exploit the female-specific market.**

We thank the reviewer for pushing us to think more about the relationships between gender, health, and medicine. However, as we describe further below in response to the reviewer's next comment, our analyses were limited by only being able to consider reporting categories that are part of the US FDA DTSP reports. Unfortunately, these reports currently only capture binary sex data and no data on gender.

The paper by Lee et al. analyzes post-marketing adverse event reporting but not disease incidence or prevalence. Although the paper is methodologically distinct from our manuscript in the sense that different endpoints are used, we did now incorporate the paper's broader point—that women and men interact with the health system differently—in the **Discussion**: **LINE 430-440**: *Second, both prevalence and incidence data can be affected by patients' behavioral patterns. For instance, it is argued there are healthcare utilization differences between the sexes that may complicate interpretation of these data[63]. Women's greater frequency of care-seeking may inflate estimates of disease prevalence. Conversely, diagnostic frameworks built on male phenotypes systematically under-recognize certain diseases in women. This paradox is well documented for cardiovascular diseases, where women are sometimes*

diagnosed years later than men despite presenting with equivalent symptoms[64]. Such delays could likely lead a woman with a health issue to seek healthcare solutions at a higher frequency until the disease is properly diagnosed and treated. Similar patterns exist for endometriosis and inflammatory bowel disease, where misdiagnoses and delays in diagnoses disproportionately affect women[52,64–66]. Improved diagnostic accuracy and consistent measurement frameworks will contribute to improved insights in disease burdens.

Our Discussion section (LINE-198-464) expands further on the reason for differences - showing that the dynamics extend well beyond women's higher use of the health-care system.

Regarding a "cynical trend" to create female-specific diagnoses
We find no empirical evidence that our results are driven by such a trend. Aligning market opportunity with genuine unmet medical need—particularly in women's health—can be a constructive pathway for innovation rather than inherently cynical. The literature cited by the reviewer appears to represent sociological critiques rather than evidence specific to our dataset.

If the reviewer is referring to (i) **biomedicalization of women's bodies** (e.g., Clarke; Martin; Ehrenreich) and (ii) **disease mongering** (Moynihan et al., 2002), then we wish to note that such dynamics are not unique to women; analogous critiques exist in literature on men's health as well.

Equally problematic is the out of date framing of the study benchmarks as a simple binary comparison between the representation of women and men in medical science. In studies of gender and health in the US context it is vital and cardinal to include subgroup analysis within these categories (eg, Krieger 2018, "US black and white women born 1915-1979") because the "equal" representation of men and women can obscure significant distortions in the representation of particular subgroups of men and of women that would cause us to revise our understanding of gendered disparities in inclusion and representation in medical science.

We appreciate this important point and fully agree that intersectional subgroup analyses are critical for uncovering deeper disparities. Our dataset was limited to reporting categories that are part of the US FDA DTSP reports, which currently capture only binary sex data.

We are acutely aware of the importance of ancestral differences. In fact, Dr. Zaaijer has written on this topic in Cell and has investigated the inclusion of patient ancestry as a factor in clinical trial design (Accepted in Communications Medicine – currently in press). However, the FDA does not publicly report ancestral data disaggregated by sex—a limitation also noted in a recent study by Bierer: Bierer BE, Meloney LG, Ahmed HR, White SA. Advancing the inclusion of underrepresented women in clinical research. Cell Rep Med. 2022 PMID: 35492242. Who writes about FDA data: "*while sex and race are reported, the interaction of sex and race are not, and thus the data cannot generally be analyzed by intersectional characteristics.*"

Looking ahead, we strongly support the inclusion of intersectional demographic frameworks (e.g., race, age, income, gender identity) as data systems evolve and become more granular.

We also acknowledge that the FDA's current reporting structure reflects the challenge of balancing detailed subgroup analyses with sufficient statistical power. This trade-off will require continued attention as trial reporting standards mature.

In summary, while deeper intersectional analyses are beyond the scope of this study, we share the reviewer's perspective. Our goal is to highlight both the progress made and the limitations that remain, providing a foundation for future work to expand and refine this effort.

Thus, while I think the empirical work undertaken in this study has potential to add to the literature, it is not publication-ready and needs significant rethinking and contextualization in the appropriate literature to ensure that appropriate inferences are made from the authors' findings.

Reviewer #2 (Remarks to the Author):

Thank you for giving me the opportunity to review this interesting manuscript by Zaaijer and Groen. The authors analyzed 187 FDA approved drugs to investigate potential sex/gender biases in allocation of approval and in representation of female participants in the studies. According to their classification they identify an increase in trials for female-predominant conditions but a persistence of enrollment disparities.

Overall, this is an important and timely analysis that addresses a persistent health disparity. The numbers are interesting, but I need to note a number of potential sources of skewed representation given the classifications employed. I would urge the authors to at least clarify many of these point in the text, if not correct for them in the analysis.

We sincerely thank the reviewer for their thoughtful and encouraging feedback, which has meaningfully improved the quality and clarity of this manuscript. We deeply appreciate the time, care, and expertise the reviewer brought to the review process.

We are truly delighted that the reviewer found the data overview exciting—this enthusiasm reinforces the importance of making these insights widely accessible.

We hope the reviewer will be pleased with the manuscript revisions and the responses we now provide.

1. While it appears laudable that more attention is being devoted to female-predominant conditions, this group is quite heterogeneous in the current analysis. The authors group gynecological conditions

(which by definition will rarely include gender-diverse individuals in their trials and do, hence, often exclusively include biologically female participants) and lump them together with non-gynecological conditions, where a selective choice to include female participants in adequate numbers has to be made.

For the sake of clarity, it would be important to differentiate between e.g. treatment for vulvovaginal candidiasis, endometrial and prostate cancer, or endometriosis and diseases that affect both female and male patients such as multiple sclerosis. The mechanisms of inclusion for these conditions are very different and rely on different approaches by the investigator, and hence, these should be reported separately.

We appreciate the reviewer's suggestion. We added to the results and discussion for both scientific questions sections specifically regarding sex-specific treatments.

For example – LINE 106-109: "*. Among 195 FDA-approved drugs, 12 targeted gynecological conditions and four addressed andrological indications, such as prostate cancer (**Fig. 3**). In addition, 11 new drugs were indicated for subtypes of breast cancer, a disease occurring among women in 99% of cases.*"

In **Figure 3**: a specific zoom in to the point which ones are gynecological indications.

Discussion: LINE 172-178: *When stratified by prevalence among the sexes, indications with a markedly higher female relative to male burden were targeted twice as frequently as male-predominant conditions in corresponding prevalence ranges (1.5 times as frequently when removing sex-specific indications). These results answer our first question:…"*

Also in the **Results**, regarding enrollment alignment LINE: 142-145: "Seventeen trials evaluated treatments for sex-specific cancers (prostate, breast, ovarian, cervical), where enrollment inherently mirrors disease prevalence. Among the 12 trials testing treatments for non-sex-specific cancers, 10 showed enrollment patterns that were aligned with disease prevalence.

However, for **our first research question**, we deliberately included gynecological diseases in our parallel analysis to assess attention and investment across female-specific, female-predominant, and sex-neutral conditions. This broader framing reflects our goal of understanding how biomedicine addresses the full range of women's health needs.

Endometriosis, for example, affects 190 million women globally (6.5 million in the U.S.) but has long been under-recognized and under-treated. Early therapies like danazol offered limited relief and carried serious side effects. Recent FDA approvals of leuprolide (2012), Orilissa (2018), and Myfembree (2022) represent long-overdue, safer, and more individualized alternatives—marking a shift toward precision medicine and greater patient choice. These type of investments we want to highlight in addition to the others.

2. The indicated prevalence rates of the conditions appear very US-skewed. This is especially obvious in the case of HIV, which is declared a significantly male-dominated diseases. This might be true in the US, but does definitely not apply in the same manner to the prevalence of HIV at a global level. Since FDA approval is relevant for the US, but many of these conditions have global impacts, global prevalence should probably be used to define their sex-specific distributions.

This is an important point. We recognize this limitation and clarify in the **Methods** we prioritized US-based epidemiologic data to reduce heterogeneity: LINES 520-521
"*We estimated sex-specific disease burden via structured searches prioritizing U.S. data where possible (CDC/SEER)* "

and **Discussion**: LINES 442-450

*Third, disease prevalence and incidence estimates vary by geography and population. For example, women comprise 53% of people living with HIV globally. However, in sub-Saharan Africa 63% of new infections are found among women, whereas in the US women represent 19% of new cases[67,68]. Similarly, in the US 82% of newly diagnosed osteoporosis patients are women, but this falls to 62% when diagnoses are assessed globally[69]. A complicating factor for comparing disease prevalence and incidence estimates across studies is that the term "global" reflects different subsections of countries around the world. Because of the variability in which populations were included for estimating each of the global prevalence and incidence statistics, we used US-based prevalence and incidence statistics where possible (**Supplemental Table 1**).*

While global impact is acknowledged, our primary goal was to analyze **FDA decision-making trends**, which necessitated a US-centric dataset.

3. The reported from line 65 on should account for some of the skewed distributions. E.g. of the 13 studies reported for female-predominant cancers, 11 alone are for breast cancer. The current depiction does not clearly point this out, although it is an important aspect to consider when placing the numbers into a larger context. Overrepresentation of some indications in the list should be clearly stated, as this gives insight into funding, testing and approval patterns.

We agree with the reviewer and have now made this more explicit. In LINES 106-109

"*Among 195 FDA-approved drugs, 12 targeted gynecological conditions and four addressed andrological indications, such as prostate cancer (**Fig. 3**). In addition, 11 new drugs were indicated for subtypes of breast cancer, a disease occurring among women in 99% of cases*."

4. Last, I like the graphical representation, however, I am left to wonder again, if the number should not be corrected for trials that by definition do only include female or male participants. These could be

shown in the figure, but could possibly be removed from the overall calculation, as they are an outlier compared to the general distribution. An alternative would be to compare the numbers with their inclusion and without, giving a clear visual of the effect of these one-sex studies on the general analysis.

We thank the reviewer for this thought. Just to make sure: each dot (approved drug) is an independent computation. There is no relationship between the dots other than that they are plotted along the same axis. There is therefore no “overall” calculation, or influence of “outliers”.

Methods – LINES 558-563; “*For each treatment in our panel, we computed if representation of women and men in clinical trials is representative for the prevalence of the disease targeted by the treatment among the sexes as determined by chi-squared testing. Our chi-squared tests were two-sided and used a significance threshold of P < 0.05. A significant outcome indicated that the male/female ratio in a trial was not representative compared to the prevalence of a disease indication among the sexes in the US population.”*

We confirm that **Figure 3** explicitly notes single-sex trials in **Box A and Box B**.

Analysis of 195 US FDA-Approved Drugs (2015-2023) across 98 Unique Indications Demonstrates the Level of Industry's Attention to Women's Health

*Sophie Zaaijer[1,2] *, Simon C. Groen[2*]*

[1]Division of Hematology and Oncology, Department of Medicine, University of California Irvine School of Medicine, Irvine, CA, USA

[2]Institute for Integrative Genome Biology, Department of Nematology, University of California Riverside, Riverside, CA, USA;

REVIEWER COMMENTS

Addressed by Sophie Zaaijer and Simon Cornelis Groen

Reviewer #2 (Remarks to the Author):

Thanks for giving me the opportunity to re-review this manuscript by Zaaijer and Groen. I feel this extended version allows for more context and nuance and significantly improves the manuscript correcting some of the simplifications present in the short version. The authors have done a good job in placing the results in a broader – and dynamic – context and have expanded on the figures providing a more detailed and precise representation of the results.

Furthermore, I think the reviewer comments have been addressed and productively incorporated.

We thank the reviewer for the careful re-evaluation of our manuscript and for the positive feedback. We are pleased that the extended version of the manuscript was helpful in providing additional context and nuance. We also appreciate the reviewer's recognition that the previous comments have been constructively addressed and incorporated. The reviewer's insights helped us improve the clarity and presentation of the work.

I only have two minor points:

From line 180 on, in the discussion of breast vs prostate cancer, the element of mean age at diagnosis and diagnostic spread is not taken into consideration. Breast cancer is diagnosed on average at 62 years and prostate cancer at 67 years, in addition to this difference the spread of these diagnoses is somewhat wider for breast cancer with a significant number of younger patients being diagnosed who profit immensely from early detection and therapy. The current phrasing could be interpreted as if the mayor investment in breast cancer research was somewhat unjustified compared to prostate cancer research. Maybe this point could be added and the whole paragraph potentially slightly adapted.

We thank the reviewer for this helpful observation. We agree that the age distribution at diagnosis—particularly the broader spread observed in breast cancer and the presence of younger patients who benefit substantially from early detection and treatment—is an important nuance. To address this point and avoid potential misinterpretation, we have revised the paragraph to clarify that although breast cancer includes a wider diagnostic age range, most cases still occur at older ages. We now explicitly acknowledge this distribution while maintaining the intended point regarding the implications of age-related safety restrictions for clinical trial recruitment. The revised text has been incorporated in the

Discussion:

LINE 248-255

"

The third and final factor is the typically greater patient age at onset of oncological diseases. The median age at cancer diagnosis is ~67 years (~63 years for breast cancer)[21]. Although the age distribution at diagnosis—particularly for breast cancer—can show a wide spread, with a meaningful proportion of younger patients, the majority of cases still occur at older ages. Consequently, safety restrictions related to patients of reproductive age (e.g., pregnancy exclusions, contraception requirements, and teratogenic risks) are generally less limiting for conducting drug trials in oncology. For example, trials for breast cancer therapies can recruit participants among post-menopausal women, the group that carries the largest burden of this disease[21].

"

For figure 2 – what is "cancer associated"?

We thank the reviewer for pointing out that the "*Cancer-associated*" therapeutic area required clarification. Because this is a non-standard grouping, we have added an explicit explanation in the legend for Figure 2. In addition, we clarified the definition of this therapeutic area in the Methods section. The area includes indications related to complications of cancer treatment, such as chemotherapy-induced emesis, chemotherapy-associated nausea and vomiting, and chemotherapy-induced myelosuppression. A complete list of indications included in this therapeutic area is provided in Supplementary Table 2.

Improved Figure legend text:line 600-605

"The area *Cancer-Associated* includes indications such as Chemotherapy-induced emesis, Nausea and vomiting associated with chemotherapy, and Chemotherapy-induced myelosuppression"

Improved Methods section text: line 515-518

"

In addition, a separate therapeutic area, *Cancer-associated*, was created to capture indications related to complications of cancer treatment. This category includes chemotherapy-induced emesis, chemotherapy-associated nausea and vomiting, and chemotherapy-induced myelosuppression. "

Reviewer #3 (Remarks to the Author):

Thank you for the opportunity to review the manuscript by Zaaijer and Groen. The research addresses an important and timely question; however, I have substantial concerns regarding the methodological approach to data analysis and interpretation.

We thank the reviewer for the careful evaluation of our manuscript and for highlighting important methodological and interpretive considerations. We appreciate the opportunity to further strengthen our manuscript through this review process. In response to the points raised, we have revised the manuscript to clarify our analytical framework, refine the interpretation of our results, and improve transparency regarding methodological choices and limitations. We address each of the reviewer's comments in detail below and believe that these revisions have substantially improved the clarity and rigor of the study. We are grateful for the reviewer's thoughtful feedback.

1. Data characterization

I find the analyses presented in the "Data Characterization" section and Figure 2 (with supporting Table S2-1) to be methodologically problematic. The authors should define the studied conditions that permits meaningful clinical interpretation of sex-based differences. For example, gynecological conditions should be excluded, as the study population consists exclusively of women by design; their inclusion alongside sex-agnostic conditions such as hyposexuality conflates distinct phenomena and obscures interpretability. Similarly, sex-predominant malignancies—including prostate, breast, ovarian, and cervical cancers—should be stratified separately or excluded to enable readers to draw valid inferences about disease representation. I recommend applying this stratification principle systematically across all reported analyses.

We thank the reviewer for this helpful suggestion. Our intention in the "Data Characterization" section was to illustrate the range of sex-based disease prevalence across the indications represented in our dataset. We agree that combining sex-specific indications (e.g., gynecological diseases or prostate cancer) with conditions affecting both sexes can complicate interpretation when evaluating sex-based representation.

We therefore revised sections in the manuscript to explicitly distinguish sex-specific indications from non-sex-specific indications.

We re-categorized the hyposexuality under neurology – given the indications are targeting neurological pathways.

The first central question we split into sex-specific, and sex-predominant. Next for the evaluation if enrollment was according to the burden – we removed the 27 sex-specific trials

from the analysis (from 218 → 191 trials remain). In disease-area analysis we removed the "reproduction" category.

For the first analysis, the distinction is described here: line 126-133

For the second analysis on trial inclusion, the distinction is described here: (line 118-126)
"

To address our second question, we tested whether women's enrollment in late-stage (pivotal) trials reflected their disease burden at the indication level. Using sex-disaggregated enrollment data from US FDA DTSP reports, we compared each trial cohort's sex ratio with our indication-specific relative prevalence ratio. For this analysis, we excluded 27 sex-specific indications, leaving 191 trial cohorts. Among these cohorts, female inclusion did not differ from the estimated sex-based prevalence of the targeted indication in 43.5% (n=83) of cases (χ^2 test, Benjamini-Hochberg adjusted P>0.05"

In the analysis per therapeutic area, the distinction is further described for indications in the *Cancer* area (line139-145):
"

We analyzed sex-based enrollment patterns by therapeutic area (Fig. 5) to assess if biases existed for certain disease types. For *Cancer* indications, seventeen trials evaluated treatments for sex-specific cancers (prostate, breast, ovarian, cervical), where enrollment inherently mirrors disease prevalence. Among the 12 trials that tested treatments for non-sex-specific cancers, 83% (n= 10) showed enrollment patterns that were aligned with disease prevalence, one over-enrolled women, and the other over-enrolled men (Fig. 5). .

"

Finally, we have updated the "Data Characterization" section as follows (line 89-101):
"

We analyzed a total of 98 specific disease indications (falling under 14 therapeutic areas) for which drugs were developed that attained FDA approval between 2015 and 2023 (Fig. 1). Each therapeutic area showed a wide variation in disease prevalence ratios between men and women (as derived from disease prevalence and incidence data, see Methods) across individual indications (Fig. 2, Table S2-1). Sex-specific cancer indications (e.g., prostate,

breast, ovarian, and cervical cancer) are categorized under the therapeutic area *Cancer* and are included to characterize the broader landscape of attention, even though they do not permit direct comparison of representation in clinical trials between the sexes."

2. Statistical testing and multiple comparisons

The use of chi-squared tests at a nominal significance threshold ($p < 0.05$) to assess sex representation within clinical trials against disease prevalence is potentially problematic given the number of hypothesis tests conducted in this study. The authors should address multiple hypothesis testing through rigorous correction methods such as Bonferroni adjustment, the Benjamini-Hochberg procedure, or the Holm method, rather than proceeding without correction. This is important for controlling the family-wise error rate and ensuring the reliability of reported findings.

This is an important point that the reviewer raises and we fully agree with the need for multiple testing correction. We have now implemented the Benjamini-Hochberg procedure to correct the P values from the chi-squared tests.

Implemented throughout the manuscript, also in the Supplemental table 4.

3. The use of LLM for data standardization

I have concerns about the reliance on Perplexity AI's natural language processing for data standardization. While LLM-based preprocessing may serve as a valuable initial step, the approach requires comprehensive human (manual) validation to ensure accuracy and fidelity. Large language models may exhibit variable performance across different clinical conditions—performing reliably for certain diagnoses while potentially underperforming for others—which risks introducing systematic bias into the dataset and downstream conclusions.

We thank the reviewer for raising this important point regarding the use of large language models (LLMs) in data standardization. In our workflow, the LLM was used only as an initial classification step to map drug approvals to broad disease categories. All assignments were subsequently validated through manual review to ensure accuracy and consistency.

To assess reliability, we implemented an internal validation step using approvals for which the FDA already provided disease annotations. Specifically, we compared LLM-derived

assignments with FDA annotations for 129 approvals and observed 97% concordance (125/129). This high agreement provided confidence in the automated categorization step.

For the remaining approvals that lacked FDA disease annotations (n = 85), both authors independently reviewed and verified the assignments manually, ensuring accuracy and consistency of vocabulary used prior to inclusion in the dataset and downstream analyses.

We have clarified this workflow in the Methods section (Lines 501–502) to better describe the role of the LLM and the extent of manual validation.

Line 502-513

"

To assess the reliability of this step, PPX-derived assignments were compared with FDA-provided disease annotations for approvals where these were available (n=129). Concordance was high (125/129; ~97%). For approvals lacking FDA disease annotations (n=85), both authors independently reviewed the assignments to ensure accuracy and vocabulary consistency prior to inclusion in the dataset and analysis.

"

The revision shows that the authors have substantially strengthened several aspects of the manuscript. The expanded Discussion, which now addresses issues such as portfolio focus, enrollment proportionality, reproductive risks, endpoint and diagnostic complexity, and sponsorship and oversight, goes a long way toward remedying the original lack of contextualization within the literature on women's health and clinical trial representation. The change from an arbitrary 75% prevalence cutoff to a standard deviation–based classification around the US Census mean is also a clear methodological improvement that better reflects variation in sex-specific disease burden across indications.

However, some of the core conceptual concerns raised by the reviewer remain only partially resolved.

A central issue is the question the reviewer posed about what should properly count as progress in commitment to women's health, and what benchmarks are appropriate to assess it. The authors now justify the use of prevalence and incidence more carefully and give a cogent rationale for using prevalence as the denominator for chronic conditions such as ADRD, where prevalence better captures the pool of patients eligible for trials.

Yet the manuscript still tends to conflate alignment of enrollment with sex-disaggregated disease burden, within a binary sex framework, with equity or commitment. Proportional enrollment is a useful metric, but it is only one possible benchmark and it is sensitive to the diagnostic and healthcare utilization processes that generate prevalence estimates in the first place.

The authors do acknowledge that women's greater healthcare utilization can inflate prevalence and that male-phenotype-based diagnostic criteria can under-recognize disease in women, but they stop short of drawing out the full implication: **that their chosen benchmark is itself shaped by gendered social and clinical practices, and that "alignment with prevalence" cannot be treated as a straightforward measure of fairness or equity**. A more explicit reflection on why this particular benchmark was chosen, what its limitations are as a measure of progress, and how it might diverge from other plausible benchmarks would strengthen the conceptual core of the paper.

We thank the reviewer for raising this important conceptual point. We agree that alignment of trial enrollment with sex-disaggregated disease prevalence should not be interpreted as a definitive measure of equity, since prevalence estimates themselves can be influenced by diagnostic practices, healthcare utilization patterns, and gendered clinical frameworks.

Our objective in this study, however, is more limited. Epidemiological benchmarks—such as prevalence estimates from SEER—are widely used within the biomedical ecosystem to characterize disease burden and inform trial design and recruitment strategies. Clinical trials are therefore already implicitly anchored to these epidemiological estimates.

In this context, our analysis asks whether trial enrollment reflects the same epidemiological distributions that the field itself relies on when defining disease burden and designing studies. In other words, we evaluate whether these existing benchmarks are being operationalized in practice.

We now clarify in the manuscript that prevalence-based proportional enrollment should be interpreted as one pragmatic benchmark within the current research framework rather than a definitive measure of equity. We also added text acknowledging that epidemiological estimates themselves may reflect gendered diagnostic and healthcare utilization patterns, and that future work will need to refine these benchmarks as understanding of sex and gender differences evolves.

Line 437-444

"

Second, epidemiological estimates themselves may be influenced by healthcare utilization patterns and diagnostic frameworks. Women generally seek healthcare more frequently than men[63], which may inflate prevalence estimates in some contexts. Conversely, diagnostic criteria historically calibrated on male phenotypes can underrecognize disease in women, as documented for *Cardiovascular Diseases*[64] and several chronic conditions[52,64–66]. As a result, epidemiological benchmarks—such as those derived from SEER and related datasets—should not be interpreted as definitive measures of equity. Instead, we use them as pragmatic reference points anchored in the same epidemiological data that commonly inform clinical trial design and disease burden estimates in biomedical research.

"

On incidence versus prevalence, the authors' revision is a clear improvement. They now distinguish thoughtfully between incidence and prevalence, invoke CDC definitions, and treat chronic versus acute conditions differently. The justification for using prevalence for ADRD, in my view, is reasonable. Nonetheless, because the reviewer's concern was not merely technical but tied to broader demographic and age-structure issues, it would help if the manuscript more openly acknowledged that different choices of denominator can yield different pictures of burden and representation and that what counts as appropriate depends on the normative question one is asking. The methodological decision to use prevalence for certain conditions should be explicitly connected back to the conceptual question of what commitment and progress mean in this

context.

We thank the reviewer for this helpful suggestion. We agree that the choice of denominator (incidence versus prevalence) can yield different perspectives on disease burden and representation, and that the appropriate metric depends on the conceptual question being asked. In this study, our goal was to evaluate whether clinical trial enrollment patterns reflect the composition of the population of people living with a disease and who are therefore potentially eligible for certain treatments. For chronic conditions such as ADRD, prevalence provides the most appropriate benchmark for this question. We now clarify this point explicitly in the manuscript and added a paragraph to the Limitations section acknowledging that different denominators address different normative questions and may produce different perspectives on representation.

Line 429-436

"

Several limitations of our analyses warrant discussion. First, our estimates of sex-specific disease burden draw on both prevalence and incidence data, which capture different aspects of disease epidemiology. Chronic conditions are typically measured using prevalence, reflecting the population currently living with disease, whereas acute conditions are better captured by incidence, reflecting new cases over time[62]. Because these metrics are not directly comparable, our analyses rely on sex ratios to mitigate potential biases when benchmarking trial enrollment against disease burden. Nevertheless, different denominators address different normative questions and may produce different perspectives on representation.

"

The question of intersectionality is only partially addressed. The authors point out that the FDA DTSP data structure allows only binary sex and that sex-by-race interactions are not reported, and it cites the Bierer paper to show that this is a recognized limitation. However, the manuscript itself does not adequately convey how serious a conceptual limitation this is for any claims about equity.

The analyses aggregate all women into a single category and then interpret improvements in aggregate female representation as evidence of progress. The reviewer's point was that such aggregation can "obscure significant distortions" for particular subgroups of women and men. The manuscript would benefit from explicitly stating that its binary sex analysis cannot show whether improvements apply across racial and socioeconomic subgroups, and that claims about "equity" and "commitment to women's health" should therefore be read as applying only at the most aggregated level. This limitation should be clearly flagged in the main Discussion where the authors interpret their findings, not just in the Methods or Limitations, and it should be explicitly connected to the reviewer's concern about subgroup disparities.

We thank the reviewer for highlighting the importance of intersectional perspectives in evaluating equity in clinical research. We agree that analyses based on binary sex categories alone cannot capture potential disparities across racial, ethnic, or socioeconomic subgroups. Because the FDA Drug Trials Snapshots Program reports enrollment primarily using binary sex categories and does not consistently provide sex-by-racial (or ethnic) group or socioeconomic breakdowns, our analysis necessarily aggregates all women into a single category. We have now clarified this conceptual limitation in the Discussion and explicitly note that improvements in aggregate female representation do not necessarily indicate equitable participation across all subgroups of women. Our findings should therefore be interpreted as reflecting changes in representation at the most aggregated level of the female biological sex.

Line 464-471"

Finally, our study evaluates representation using the binary sex categories reported in the FDA DTSP data. These regulatory datasets capture sex as recorded in trial documentation and do not include gender identity or consistent intersectional breakdowns (e.g., sex-by-race or socioeconomic status). Consequently, representation of gender-diverse individuals and subgroup disparities in clinical trial cohorts cannot be assessed within this framework, and improvements in overall female participation may mask differences across demographic groups. Our findings should therefore be interpreted as reflecting patterns in representation at the aggregate sex level rather than a comprehensive assessment of gender equity.

”

The framing of the paper, especially in the title and some strong statements in the Discussion, still presents the results in terms that are more definitive than the data justify. Phrases that the study “demonstrates the level of industry’s commitment to women’s health” and that industry is “meaningfully investing in lowering female disease burden” go beyond what can be established from patterns of approvals and trial enrollment alone. The reviewer’s original concern about “the right measures and benchmarks for ‘progress’ in commitment to women’s health” has not yet been fully absorbed into the framing. It would be more accurate to describe the study as assessing patterns in approvals and enrollment that are consistent with increased attention to conditions with a higher female disease burden and with more proportional inclusion of women in some areas, while also documenting areas where women remain underrepresented. The interpretive language should be recalibrated from strong claims about commitment and progress to more modest claims about what the observed distributions can and cannot tell us.

We thank the reviewer for this thoughtful comment regarding the framing of our findings. We agree that patterns in drug approvals and clinical trial enrollment cannot by themselves establish intent or fully capture the concept of “commitment” to women’s health. Our goal was to evaluate observable outcomes within the drug development system—specifically, which disease indications have received approval of therapies since 2015 and how women were represented in pivotal trials supporting those approvals. Because these decisions occur at the most consequential stage of drug development, they provide a meaningful lens into how disease burdens among women are reflected in the therapeutic pipeline.

We have revised the manuscript to clarify that our findings should be interpreted as patterns consistent with increased attention (rather than commitment) to conditions with higher female disease burden and to proportional inclusion of women in certain therapeutic areas**, while also documenting areas where women remain underrepresented. We retained a constructive interpretation of these patterns as encouraging signals within the current system, while tempering language that might be read as implying direct evidence of intent or commitment.**

Line 175-180

Focus of Drug-Development Portfolios: 1.5-5.5x Greater Emphasis on Female-Predominant Indications

Our results suggest that, since 1993, the biopharmaceutical industry has become increasingly attentive to disease areas with a higher female burden, as reflected in both approval patterns and the representation of women in pivotal clinical trials. These observations indicate that conditions affecting women receive substantial therapeutic attention within the current drug development landscape. However, progress appears to have plateaued over the past decade, and important gaps remain.

In addition, we have now changed the word "commitment" in the original title of our manuscript to "attention" in the updated revision of our manuscript.

Finally, the manuscript still tends to slide between "sex" and "gender" language, despite the authors' acknowledgment that the FDA captures binary sex and not gender identity. This was part of the reviewer's broader critique that the manuscript's framing as a binary comparison between "men" and "women" is out of date in contemporary gender and health research.

To respond fully, the authors should not only clarify terminologically that they are analyzing sex as recorded in regulatory documents, but also reflect briefly on what is missing from this picture: gender-diverse individuals, the social dimensions of gender, and intersectional positions that cannot be captured by a binary variable. This reflection should be connected back to the reviewer's point that progress in representation cannot be adequately assessed with a strictly binary framework and that the study's conclusions about "gender equity" should therefore be carefully qualified.

We fully agree with the reviewer that it is important to highlight the distinction between sex and gender in contemporary health research. Our analysis relies on data from the US FDA's Drug Trials Snapshots Program, which report participant demographics using binary sex categories (biologically male or female) and unfortunately do not capture gender identity. We have revised the manuscript to clarify that our analysis evaluates representation based on biological sex as recorded in regulatory documents. We also added a paragraph to the Discussion noting that this binary framework cannot capture gender-diverse individuals or the

broader social dimensions of gender and that our conclusions therefore apply to representation at the level of binary sex categories rather than a comprehensive assessment of gender equity.

Line 464-471"

Finally, our study evaluates representation using the binary sex categories reported in the FDA DTSP data. These regulatory datasets capture sex as recorded in trial documentation and do not include gender identity or consistent intersectional breakdowns (e.g., sex-by-race or socioeconomic status). Consequently, representation of gender-diverse individuals and subgroup disparities in clinical trial cohorts cannot be assessed within this framework, and improvements in overall female participation may mask differences across demographic groups. Our findings should therefore be interpreted as reflecting patterns in representation at the aggregate sex level rather than a comprehensive assessment of gender equity.

"

REVIEWER COMMENTS

Reviewer #2 (Remarks to the Author):

All comments have been addressed.

We thank the reviewer for their careful evaluation of our manuscript and for confirming that all comments have been addressed. We appreciate the constructive feedback provided throughout the review process, which has helped strengthen the clarity and rigor of our work.

Reviewer #3 (Remarks to the Author):

I thank the authors for their detailed responses and for the substantial effort invested in this revision. The implementation of Benjamini-Hochberg correction, the shift to a SD-based prevalence classification, the stratification of sex-specific from sex-predominant indications, the clarification of the LLM validation workflow, and the expanded discussion all represent improvements. Despite these improvements, several core conceptual concerns remain only partially resolved.

We thank the reviewer for their thoughtful evaluation and for recognizing the substantial revisions and improvements made to the manuscript. We appreciate the acknowledgment of the methodological and conceptual refinements.

We also appreciate the reviewer's continued engagement with the conceptual framing of the study. In this revision, we have further strengthened the Discussion to more clearly contextualize our findings, explicitly acknowledge key limitations, and refine our interpretations to avoid overstatement. We believe these changes address the remaining concerns by more precisely delineating the scope and implications of our results, while maintaining the core contributions of the study.

First, I previously pointed out that "alignment with prevalence" cannot be treated as a straightforward measure of fairness, because the prevalence benchmarks themselves are shaped by gendered diagnostic practices and differential healthcare utilization. The authors have added an acknowledgment of this in the limitations section (lines 437–444), which I appreciate. However, the discussion continues to frame the 67% alignment finding as "an encouraging signal that suggests conscious efforts toward female inclusion" without any qualifier/justification. Most readers will engage primarily with the discussion, not the limitations, and will encounter the central finding decontextualized/misunderstood. I ask the authors to add two to three sentences directly to the discussion**, adjacent to the 67% interpretation, acknowledging that "alignment with prevalence" should be read as a pragmatic rather than normative measure of representation.**

We thank the reviewer for this important clarification. We have revised the Discussion to directly contextualize the 67% alignment finding. Specifically, we removed intent-implying language and added explicit qualifying sentences immediately following this result. These clarify that alignment with prevalence should be interpreted as a pragmatic rather than a normative benchmark, and that prevalence estimates are shaped by patterns of diagnosis and healthcare access. This ensures that the central finding is appropriately framed within the main Discussion rather than relying solely on the Limitations section.

LINES 213-220
“””
These results should be interpreted within important constraints. First, alignment to disease prevalence serves as a pragmatic benchmark rather than a definitive standard of equitable representation, as prevalence estimates are themselves shaped by patterns of diagnosis and healthcare access [...]
“””

Second, I requested that the intersectionality limitation be flagged in the main discussion where findings are interpreted, not only in limitations. The paragraph added at lines 464–471 is welcome but does not fulfill this request. Positive findings for oncology and neurology enrollment are interpreted in the discussion without any qualifier that these gains apply only at the most aggregated level of binary biological sex and may not reflect equitable participation across racial, ethnic, and socioeconomic subgroups of women. I ask for a brief qualifier, at minimum at the close of the "Enrollment Proportionality" subsection, making this clearer. The Limitations paragraph should remain, but cannot substitute for an in-Discussion acknowledgment.

We agree and have incorporated this clarification into the main Discussion. At the close of the “Enrollment Proportionality” subsection, we now explicitly state that our findings are based on aggregate comparisons using binary biological sex and may obscure disparities across racial, ethnic, and socioeconomic subgroups of women. This ensures that the interpretation of positive findings is appropriately qualified within the Discussion itself, rather than only in the Limitations section.

LINE 224-226
“””[...] Third, these findings rely on aggregate comparisons based on binary biological sex and may obscure disparities in participation across racial, ethnic, and socioeconomic subgroups of women.[...]

Third, the title change from "commitment" to "attention" is welcome. However, the phrase "an encouraging signal that suggests conscious efforts toward female inclusion" (lines 202–205) attributes deliberate industry agency to observational distributional patterns, which the study design cannot establish. I ask the authors to replace intent-implying phrases with pattern-based language throughout. For example, "patterns consistent with increased female inclusion" in place of "conscious efforts toward female inclusion." This is

a request for language precision appropriate to an observational study, not a request to weaken its message.

We have revised the manuscript throughout to remove intent-implying language and ensure consistency with the observational nature of the study. In particular, we replaced prior language suggesting deliberate efforts with neutral, pattern-based phrasing and now describe the findings as indicating partial alignment between enrollment and disease prevalence.

LINE 206-207
“””
Overall, the findings indicate partial alignment: in 67% of pivotal trials, women’s enrollment met or exceeded indication-specific relative prevalence among females…
“””””

Fourth, the discussion presents proportional enrollment as an achievement without drawing out a further implication: if the benchmark is inflated by women's higher healthcare utilization, alignment with it may represent alignment with a floor rather than a meaningful ceiling of appropriate inclusion. Conversely, where diagnostic criteria are male-calibrated, as in cardiovascular disease, "alignment" may reflect alignment with an underestimate of the true female disease burden. I ask for one to two sentences in the Discussion making this circular dependency explicit.

We now explicitly state that alignment with prevalence may reflect concordance with an elevated benchmark in contexts where prevalence is influenced by higher healthcare utilization among women. Conversely, in conditions where diagnostic criteria are historically calibrated to male presentations, alignment may reflect concordance with a lower bound of the true disease burden in women. These additions clarify the dependency between benchmark definition and interpretation directly within the Discussion.

[…] In contexts where women have higher healthcare utilization, observed prevalence may be inflated, and apparent alignment may therefore reflect concordance with an elevated benchmark rather than true proportional inclusion. Conversely, in conditions where diagnostic criteria are historically calibrated to male presentations, prevalence in women may be underestimated; in such cases, alignment may reflect concordance with a lower bound of the true disease burden. […]

Finally, despite acknowledging that the FDA DTSP captures binary biological sex rather than gender identity, the manuscript continues to intermittently use "gender" where "sex" is the correct term. A thorough, line-by-line audit is required before this manuscript can be considered terminologically consistent with its own epistemological framing.

We thank the reviewer for raising this important point. We conducted a thorough, line-by-line audit of terminology throughout the manuscript. We did not identify instances where

"gender" is used in place of "sex" when referring to the biological variable captured in the FDA DTSP dataset. The term "gender" is used only where appropriate—specifically to refer to gender identity, which is not captured in the dataset—and in cited literature. We therefore believe the manuscript is terminologically consistent with its stated epistemological framework. However, if the reviewer has identified specific instances we may have overlooked, we would greatly appreciate guidance to those locations so we can address them directly.

Where we use the word "gender", lines 484-491

"Finally, our study evaluates representation using the binary sex categories reported in the FDA DTSP data. These regulatory datasets capture sex as recorded in trial documentation and do not include gender identity or consistent intersectional breakdowns (e.g., sex-by-race or socioeconomic status). Consequently, representation of gender-diverse individuals and subgroup disparities in clinical trial cohorts cannot be assessed within this framework, and improvements in overall female participation may mask differences across demographic groups. Our findings should therefore be interpreted as reflecting patterns in representation at the aggregate sex level rather than a comprehensive assessment of gender equity."

Minor points:

1. The temporal regression ($F_{1,7}$ = 0.0142, P = 0.909) is presented as showing "no change across years," but nine annual observations provide insufficient power to support this conclusion. The finding should be reframed as "no statistically detectable trend," with an explicit acknowledgment that absence of evidence is not evidence of absence.

We have revised the text to state that no statistically detectable trend was observed, rather than implying stability over time. We further added an explicit qualification noting that the limited number of annual observations restricts statistical power, and that the absence of a detectable trend should not be interpreted as evidence of no change.

Lines 144-147
"No statistically detectable trend in the enrollment of women in clinical trials was observed over the nine years analyzed (linear regression, $F_{1,7}$ = 0.0142, P = 0.909; Supplementary Figure 1). However, given the limited number of annual observations, this finding should not be interpreted as evidence of stability over time."

2. The two osteoporosis trials enrolling 100% women are noted but not examined. For internal consistency with the paper's equity framing, the reciprocal exclusion of men, who account for approximately 18% of osteoporosis cases, warrants at least brief comment.

We thank the reviewer for highlighting this important point regarding internal consistency in our equity framing. We have now added a brief statement in the "Enrollment Proportionality" subsection of the Discussion to explicitly address this observation.

Specifically, we note that in osteoporosis trials, women comprised 100% of enrolled participants despite men accounting for approximately 18% of cases, underscoring that overrepresentation of one sex may also reflect the reciprocal exclusion of the other. This addition ensures that both underrepresentation and overrepresentation are interpreted within the same equity framework.

Lines 209-212
“””This overrepresentation represents the opposite extreme; for example, in two osteoporosis trials, women comprised 100% of participants, despite men accounting for approximately 18% of osteoporosis cases. At the same time, women are underrepresented in 33% of trials, with these disparities concentrated in specific disease areas (discussed below).
“””

3. Finally, the survivorship bias hypothesis (lines 406–425) is a material confounder for the 67% alignment claim and should be noted in the Abstract so that readers do not encounter the central finding without this important qualification.

To ensure appropriate interpretation of the 67% alignment finding, we have revised the Abstract to explicitly clarify that this estimate is based on trials supporting FDA-approved therapies. This makes clear that the finding reflects a subset of successful trials, consistent with the more detailed discussion of this limitation in the main text.

Lines 20-26:
“A 5.5-fold higher FDA approval rate for therapies addressing female-specific indications was observed. Additionally, female-predominant indications received drug approvals 1.5 times more often compared to male-predominant indications. Evaluation of enrollment only in trials that led to FDA approvals uncovered that female trial participation aligned with or exceeded relative disease prevalence in 67% of this subset of trials. Alignment was strongest in oncology, whereas cardiovascular and autoimmune/inflammatory disease trials most often under-enrolled women. No change in alignment was observed across the years.”

Point-by-point

Sex Representation in Trials Relative to Indication-Specific Disease Burden in FDA-Approved Drugs (2015–2023)

Sophie Zaaijer[1,2 *], Simon C. Groen[2*]

[1]Division of Hematology and Oncology, Department of Medicine, University of California Irvine School of Medicine, Irvine, CA, USA;

[2]Institute for Integrative Genome Biology, Department of Nematology, University of California Riverside, Riverside, CA, USA;

~~~ April 21, 2026

My second concern relates to the temporal trend analysis. The manuscript reports that no change in alignment was observed across the years and presents this as a substantive finding. However, with approximately 195 drugs approved over 9 years (roughly 22 per year), the annual sample sizes are small. As the analysis is probably under-powered, a null finding in a low-powered analysis cannot be interpreted as evidence of a true absence of temporal change. This limitation is not acknowledged anywhere in the manuscript. I ask that you either explicitly state that the temporal null finding may reflect insufficient statistical power rather than a true absence of trend, or provide a post-hoc power calculation or sensitivity analysis to support the claim.

We have revised the manuscript to explicitly acknowledge this limitation and to clarify the interpretation of the null finding.

Specifically, we now state that the absence of a statistically detectable trend over time should not be interpreted as evidence of stability but may instead reflect insufficient power given the relatively small number of approvals per year and the limited number of annual observations. We have revised the wording of the main text accordingly to avoid implying a substantive absence of change.

The revised main text now reads:

*“No statistically detectable trend in the enrollment of women in clinical trials was observed over the nine years analyzed (linear regression, $F_{1,7}$ = 0.0142, P = 0.909; Supplementary Fig. 1). However, given the limited number of annual observations, this analysis is **underpowered** and should not be interpreted as evidence of stability over time.”*

My third concern relates to the LLM validation workflow. The clarification provided is an improvement over the prior version. However, the Methods section still lacks the specificity needed for independent reproducibility. In particular, it is not reported which specific data extraction or classification tasks the LLM was applied to, how LLM outputs were validated against a human-curated ground truth, and what discordance or error rate was observed. Given that LLM-assisted data processing is not yet a standardized practice in biomedical research, this level of transparency is essential. I ask that you add a concise but complete description of these three elements.

We have substantially revised the Methods section to provide a detailed and reproducible description addressing each of the points raised.

First, we now explicitly define the LLM task as a constrained, single-label classification problem in which each drug approval was assigned to exactly one therapeutic area from a predefined, fixed set of categories. We clarify the input fields (drug name and FDA indication text), the use of a controlled category schema derived from FDA disease annotations, and the restriction that outputs must be selected from this list. To further enhance reproducibility, we include a complete example prompt and expected output, illustrating exactly how the LLM was queried and what format of response was required.

Second, we now describe in detail the validation framework. FDA-provided disease annotations (available for a subset of approvals) were mapped to the same category schema and used as an independent reference standard. Importantly, we clarify that these annotations were not used as inputs to the LLM but were used solely for validation, thereby avoiding circularity.

Third, we now report the quantitative performance of the LLM-based classification, including both concordance and discordance. Specifically, among approvals with available FDA annotations (n = 129), agreement between LLM-derived and FDA-based assignments was observed in 125 cases (96.9%), corresponding to a discordance rate of 3.1% (4/129).

Finally, we describe the post-classification quality control process, including rule-based harmonization of edge cases and independent human review with consensus adjudication for approvals lacking FDA annotations or exhibiting discordance. We emphasize that human review served as validation rather than primary classification.

We believe these revisions provide a clear, complete, and reproducible description of the LLM-assisted workflow and directly address the reviewer's concerns.

Fourth concern: We agree with the referee that the title should be moderated and remain relatively neutral in tone. While we appreciate that this point has been raised previously, it is not uncommon— during peer review and also editorially—for titles to be revised even in later rounds. As a suggestion, you may wish to consider:
"Analysis of FDA-approved drugs (2015–2023) across specific indications examines sex representation relative to disease prevalence."
Please feel free to adjust further, provided the tone remains appropriately balanced and that it does not surpass 15-16 words

The revised title now reads:

"*Sex Representation in Trials Relative to Indication-Specific Disease Burden in FDA-Approved Drugs (2015–2023)*"
